# Feeding diversified protein sources exacerbates hepatic insulin resistance via increased gut microbial branched-chain fatty acids and mTORC1 signaling in obese mice

**DOI:** 10.1038/s41467-021-23782-w

**Published:** 2021-06-07

**Authors:** Béatrice S.-Y. Choi, Noëmie Daniel, Vanessa P. Houde, Adia Ouellette, Bruno Marcotte, Thibault V. Varin, Cécile Vors, Perrine Feutry, Olga Ilkayeva, Marcus Ståhlman, Philippe St-Pierre, Fredrik Bäckhed, Angelo Tremblay, Phillip J. White, André Marette

**Affiliations:** 1 Quebec Heart and Lung Institute (IUCPQ), Université Laval, Québec, Canada; 2 Institute of Nutrition and Functional Foods (INAF), Université Laval, Québec, Canada; 3 Duke Molecular Physiology Institute and Department of Medicine, Duke University, Durham, USA; 4 Wallenberg Laboratory, University of Gothenburg, Gothenburg, Sweden; 5 Novo Nordisk Foundation Center for Basic Metabolic Research, University of Copenhagen, Copenhagen, Denmark; 6 Department of Pharmacology and Cancer Biology, Duke University, Durham, USA

**Keywords:** Microbiome, Metabolic diseases

## Abstract

Animal models of human diseases are classically fed purified diets that contain casein as the unique protein source. We show that provision of a mixed protein source mirroring that found in the western diet exacerbates diet-induced obesity and insulin resistance by potentiating hepatic mTORC1/S6K1 signaling as compared to casein alone. These effects involve alterations in gut microbiota as shown by fecal microbiota transplantation studies. The detrimental impact of the mixed protein source is also linked with early changes in microbial production of branched-chain fatty acids (BCFA) and elevated plasma and hepatic acylcarnitines, indicative of aberrant mitochondrial fatty acid oxidation. We further show that the BCFA, isobutyric and isovaleric acid, increase glucose production and activate mTORC1/S6K1 in hepatocytes. Our findings demonstrate that alteration of dietary protein source exerts a rapid and robust impact on gut microbiota and BCFA with significant consequences for the development of obesity and insulin resistance.

## Abstract (web summary)

Diet-induced changes in the microbiome have been associated with obesity. Here, using a mouse model, the authors show that a mixed protein source found in western diets exacerbates diet-induced obesity and insulin resistance by potentiating hepatic mTORC1/S6K1 signaling via microbial production of branched-chain fatty acids (BCFA).

## Introduction

Obesity is a complex disease associated with numerous comorbidities, including metabolic syndrome and cardiovascular diseases^1^. These pathologies are commonly studied via animal models fed an obesogenic diet that is rich in fat and sucrose to mimic the western diet. In these diets much attention is given to fat and carbohydrate sources as the main culprit for the development of these diseases; however, very little consideration is given to the protein source. Indeed, the vast majority of commercially purified obesogenic diets use casein from bovine milk protein as the sole source of dietary protein. Moreover, all the Nutrient Requirement guidelines^2^ for laboratory animals which serve as a frame of reference for rodent diet producers are based on casein only. This is problematic for two important reasons: (1) casein does not have the same composition as the plant and animal sources of proteins that are abundant in most human diets, and (2) casein may reduce body weight gain as compared to other protein sources, especially in its hydrolyzed form^3,4^. Furthermore, the molecular mechanisms underlying the metabolic effects of different dietary protein types remain elusive^4–8^.

Here, we show that a mixture more representative of the complex composition of dietary protein consumed by humans in western societies promotes distinct metabolic perturbations, such as increased weight gain and insulin resistance, compared to a diet containing only casein and we explore the potential role of the gut microbiota in these effects. More specifically, we show the impact of consuming mixed dietary proteins compared to casein on liver metabolism through incomplete mitochondrial oxidation of fatty acids, as well as the activation of the mTORC1/S6K1 signaling pathway. These findings highlight the importance of considering protein sources in the diet of animal models of diet-induced obesity.

## Results

### Protein source influences the obesogenic effect of high-fat high-sucrose feeding

**Fig. 1:**
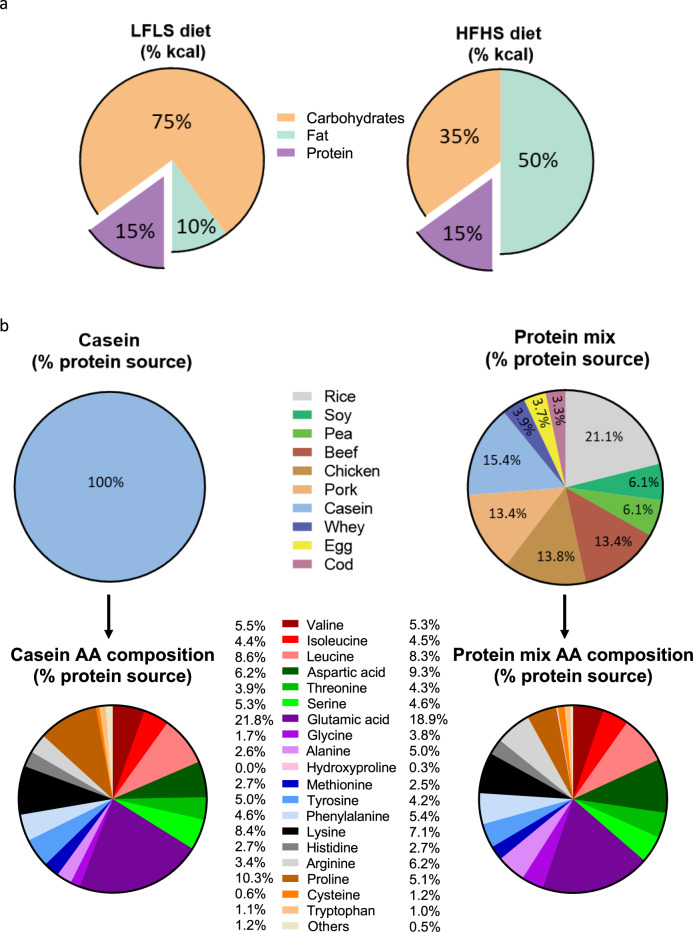
A new protein mix was formulated to oppose casein use in rodent diets. **a** Macronutrient composition of LFLS (15% kcal proteins, 10% kcal fat, 75% kcal carbohydrates) and HFHS (15% kcal proteins, 50% kcal fat, 35% kcal carbohydrates) diets. **b** Description of the protein sources used to formulate Casein (100% casein) and Protein Mix (13.4% beef, 13.4% pork, 13.8% chicken, 15.4% casein, 3.9% whey, 3.3% cod, 6.1% soy, 6.1% pea, 21.1% rice, 3.7% egg) portions in diets and resulting amino acid composition, in the percentage of protein source. See also Supplementary Tables 1–3.

We designed a protein mix (PM) that better reflects the protein composition of diets consumed by humans. This PM was incorporated into either a low-fat low-sucrose (LFLS) diet or a high-fat high-sucrose (HFHS) diet. We then compared the respective LFLS-PM and HFHS-PM diets to classical control diets which contained casein (C) as the single protein source. PM and C diets were matched for total carbohydrates, lipids, and protein amount (Fig. 1 and Supplementary Table 1). Our PM was formulated according to the USDA database^9^ recording the protein intake of the global North American population and therefore can be considered representative of the protein mixture found in a western diet. The PM included ten different protein sources: plants (rice, 21.1%; soy, 6.1%; pea; 6.1%), red (beef, 13.4%) and white (chicken, 13.8% and pork 13.4%) meats, dairy products (casein, 15.4% and whey, 3.8%), egg (3.7%) and fish (cod, 3.3%) (Fig. 1 and Supplementary Table 2). Compared to casein, the PM diet contained more than double the glycine, almost twice as much alanine, arginine, and cysteine, 50% more aspartic acid, and 50% less proline (Fig. 1b, Supplementary Table 3). The PM diet also slightly differed from casein (10–20% of variation) in the content of serine, glutamic acid, tyrosine, and lysine, which were more abundant (+11–17%), and threonine and phenylalanine (−12–16%) which were less abundant in PM compared to casein (Fig. 1b). Importantly, the diets were isonitrogenous and amino acid proportions were matched between LFLS diets and HFHS diets (Supplementary Tables 1 and 3).

**Fig. 2:**
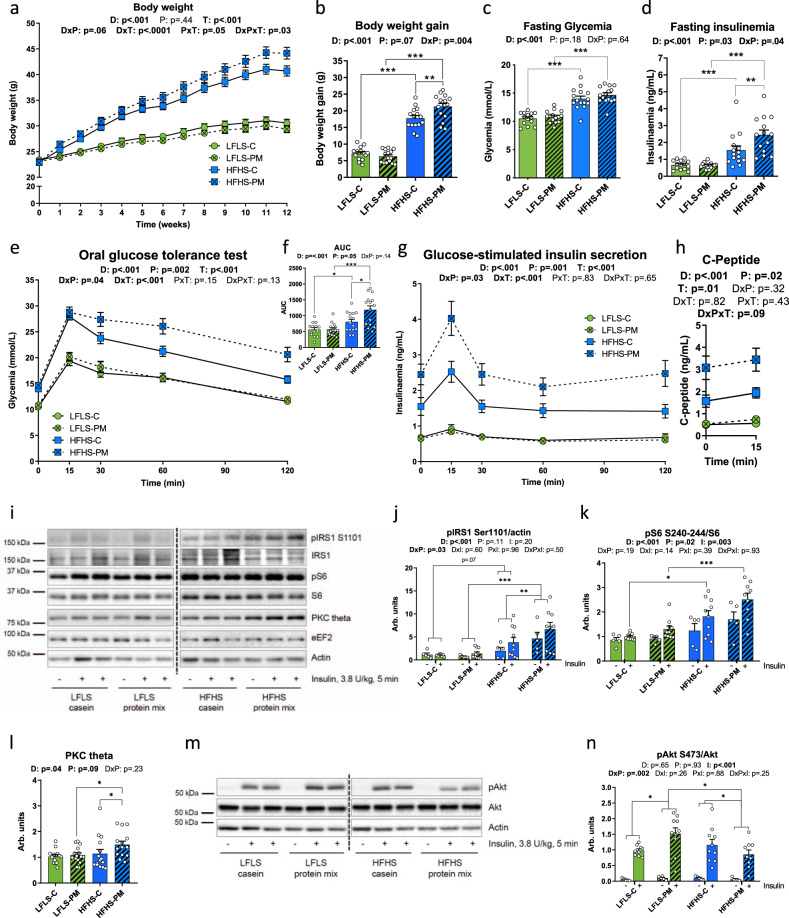
Protein mix compared to casein potentiates diet-induced obesity and glucose intolerance through mTORC1 pathway activation in the liver. Mice were fed with a LFLS-C (green), a LFLS-PM (green, hatched), a HFHS-C (blue), or a HFHS-PM (blue, hatched) diet. **a** Bodyweight curve and (**b**) total body weight gain over the 12 weeks of dietary treatment. **c**–**h** At week 11, mice were fasted for 6 h and challenged with an oral glucose load (1 mg/g body weight): (**c**) glycemia and (**d**) insulinemia before the ingestion of glucose, (**e**) glycemic response, and (**f**) area under the curve, as well as (**g**) insulinemic response following the glucose load. (*n* = 14 for LFLS-C group and *n* = 15 biologically independent mice for the three other groups except for (**d**) where *n* = 14 for HFHS-PM group). **h** C-peptide response before and 15 min after the ingestion of the bolus of glucose (*n* = 4 for LFLS-PM group, *n* = 6 for LFLS-C and HFHS-PM groups, and *n* = 7 biologically independent mice for HFHS-C group). **i**–**n** Hepatic insulin signaling of mice fasted for 6 h and injected intravenously with saline (“−“, *n* = 5 for all groups) or insulin (“+”, *n* = 9 for LFLS-C and HFHS-PM groups and *n* = 10 independent mice for LFLS-PM and HFHS-C groups) for 5 min before euthanasia: immunoblots and quantification of densitometry analyses for (**i**–**k**) pIRS1 Ser1101, total IRS1, pS6 S240-244 and total S6 (**i, l**) PKC theta (*n* = 14 for LFLS-C and HFHS-PM groups and *n* = 15 biologically independent mice for LFLS-PM and HFHS-C groups) and (**m, n**) pAkt Ser473 and total Akt. Actin and eEF2 have been used as loading controls. Representative images from different gels and separated by a dashed line. As twice as many mice were injected with insulin compared to saline, the number of representative animals (2:1) is pictured on the gels. Arb. units, Arbitrary Units. Data are represented as means ± s.e.m. Statistical analyses were performed using a two-way ANOVA, a three-way ANOVA or a mixed model for repeated measures, followed by a Tukey post-hoc test. *P*-values of general effect for diet (D), protein (P), time (T), and insulin condition (I) factors are recorded under the title of each graph, followed by the *p*-values of the corresponding factor interaction effects. Detailed significant differences detected by post-hoc test are recorded as follows: *p < 0.05, **p < 0.01, ***p < 0.001. Exact *p*-values for trends (0.05 ≤ *p*-value < 0.10) are recorded on graphs for an additional indication. See also Supplementary Figs. 1 and 2.

The protein source did not influence food intake or weight gain in mice fed the LFLS diet. However, PM amplified the obesogenic effects of HFHS feeding on body weight gain over a 12-week period (Fig. 2a). This occurred without changes in energy intake and protein consumption (Supplementary Fig 1a, b). The greater weight gain observed in HFHS-PM-fed mice compared to their HFHS-C-fed counterparts (Fig. 2b) was due to an increase in both fat mass and lean mass (Supplementary Fig. 1c, d) as evidenced by body composition analysis, as well as the increased mass of visceral adipose tissue, brown adipose tissue, and gastrocnemius muscle (Supplementary Fig. 1e-h).

Further examination of brown adipose tissue (BAT) revealed that PM downregulated the expression of several thermogenic genes in obese mice. Notably, uncoupling protein 1 (*Ucp1*), Cell death activator CIDE-A (*Cidea*), and Type II iodothyronine deiodinase (*Dio2*) mRNA levels were markedly lower in BAT of mice fed the HFHS-PM diet compared to HFHS-C fed mice (Supplementary Fig. 1i–k). In contrast, PPAR-γ-co-activator 1α (*Pgc1α*) gene expression was mainly affected by dietary fat and sucrose rather than protein source (Supplementary Fig. 1l). This suggests that lower BAT thermogenic activity may have contributed to the higher weight gain in mice fed the HFHS-PM diet.

The PM diet also exacerbated the detrimental effect of HFHS feeding on glucose homeostasis. Mice fed the HFHS-PM diet displayed similar fasting glycemia but higher fasting insulinemia and larger glucose and insulin excursions during an oral glucose tolerance test compared to HFHS-C-fed mice (Fig. 2c–g). The increased insulin response likely reflects higher glucose-stimulated insulin secretion and not lower clearance of the hormone given that C-peptide levels were also increased during the OGTT (Fig. 2h) and that this beta-cell product has negligible extraction by the liver. In the LFLS fed mice, the PM had no effect on glucose homeostasis which is in line with the lack of effect on body weight gain.

Given that dietary protein and amino acids can impair insulin signaling through mammalian target of rapamycin complex 1/S6 kinase 1 (mTORC1/S6K1) dependent phosphorylation of insulin receptor substrate 1 (IRS1) on multiple inhibitory serines residues^10–14^, we next tested whether our PM diet impacts metabolic health via this nutrient-sensing mechanism. Consistent with higher mTORC1/S6K1 signaling in the liver of HFHS-PM fed mice, we observed higher inhibitory phosphorylation of IRS1 on serine 1101 concomitant with increased phosphorylation of S6 on Ser240-244 compared to HFHS-C mice (Fig. 2i–k). In addition to higher S6 phosphorylation, PKC theta, an alternative IRS-1 S1101 kinase activated by diacylglycerol (DAG) and associated with type 2 diabetes^15,16^, was also increased in the liver of HFHS-PM-fed mice as compared to HFHS-C-fed mice (Fig. 2i, l). Importantly, the net result of these effects in HFHS-PM fed mice was significantly lower insulin-mediated phosphorylation of Akt on serine 473 compared to their HFHS-C fed counterparts (Fig. 2m, n). IRS1 and IRS2 protein expression were not modulated by the protein source (Fig. 2i, Supplementary Fig. 2a–c). While the mixed protein source clearly potentiated HFHS mediated inhibitory phosphorylation of IRS1 in the liver, the addition of PM to the HFHS diet had no impact on insulin signaling in skeletal muscle (Supplementary Fig. 2d–j), suggesting that the mixed protein primarily caused hepatic rather than peripheral insulin resistance, thus PM likely exerts its effect on glucose tolerance by promoting hepatic glucose production rather than lowering glucose uptake in peripheral tissues.

### Protein source modulates gut microbiota independent of dietary fat and carbohydrate

Diet is a key environmental factor shaping the gut microbiota^17^. To evaluate the extent to which different protein sources influence bacterial populations, 16S rRNA gene sequencing was performed on DNA extracted from fresh fecal samples collected after 11 weeks of dietary treatment. Examination of the overall bacterial composition notably revealed a drastic reduction of Verrucomicrobiales by PM and blooming of Lactobacillales by HFHS and PM interaction (Supplementary Fig. 3a). Among the 30 genera identified by taxonomic assignment recorded in the heatmap, some were only present in the HFHS context like *Romboutsia* or seemed to be particularly enhanced by the PM like *Intestinimonas* (Supplementary Fig. 3b). PCoA analysis showed a clear diet-specific clustering of microbiota from LFLS and HFHS mice, as well as a tendency for the two HFHS groups to cluster separately (Supplementary Fig. 3c). As expected, the HFHS diet modulated the relative abundance of several bacterial genera and promoted *Romboutsia*, *Adlercreutzia,* and *Tyzzerella* while reducing *Bifidobacterium*, *Facecalibacterium,* and a *Muribaculaceae* genus (Supplementary Fig. 4a–d).

**Fig. 3:**
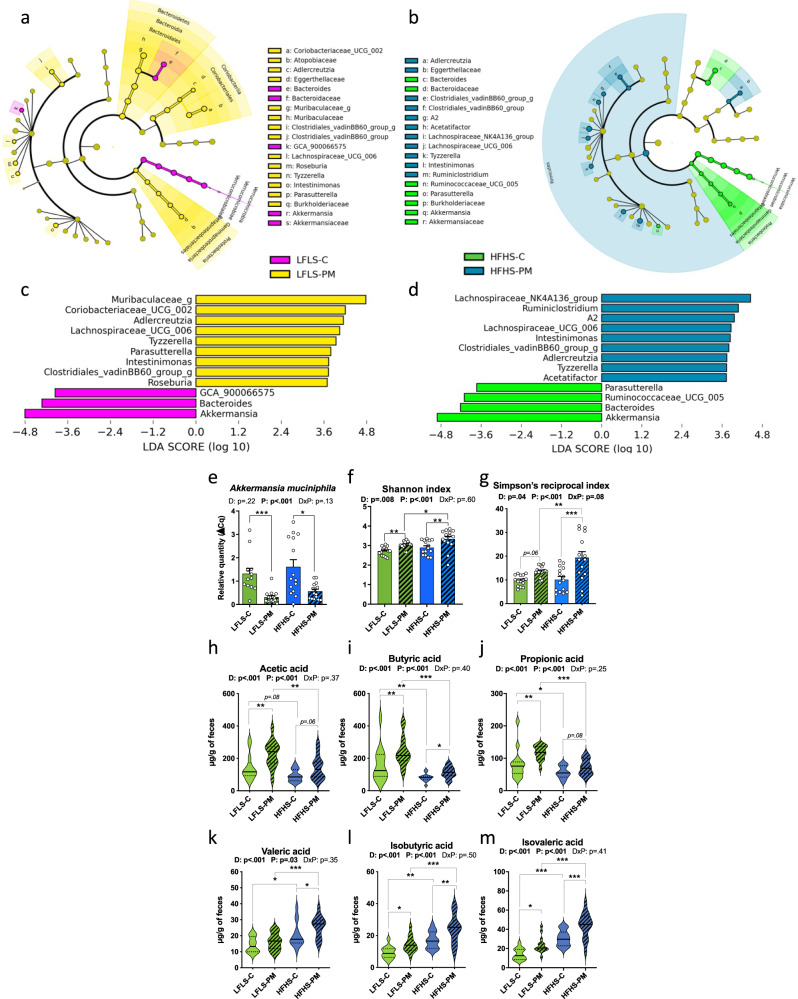
Protein mix shifts gut microbiota composition independently of LFLS or HFHS diet after 12 weeks of dietary treatment. **a**, **b** Cladograms showing differentially abundant bacteria between (**a**) LFLS-C (pink) and LFLS-PM (yellow) groups and (**b**) HFHS-C (green) and HFHS-PM (blue) groups, represented as the taxonomic levels from phylum to order, and abbreviations for family and genus levels. The central point denotes the root of the tree of bacteria. The size of each node represents the relative abundance of taxa. **c**, **d** Histograms of LDA scores identifying genera differentially represented between (**a**) LFLS-C and LFLS-PM and (**b**) HFHS-C and HFHS-PM groups, with a cut-off value of 2.5 for LDA score. The presence of ‘g’ at the end of taxon denotes an unclassified genus. **e** Relative quantity of fecal *Akkermansia muciniphila* evaluated by qPCR. Alpha diversity represented by (**f**) Shannon and (**g**) Simpson’s reciprocal indexes. (*n* = 14 for the LFLS-C group and *n* = 15 biologically independent mice for the three other groups). Fecal levels of major SCFA (**h**) acetic, (**i**) butyric and (**j**) propionic acids and minor SCFA (**k**) valeric, (**l**) isobutyric, and (**m**) isovaleric acid after 12 weeks of dietary intervention (*n* = 14 for LFLS-C and HFHS-C groups and *n* = 15 biologically independent mice for LFLS-PM and HFHS-PM groups). For panels **e**–**m**: LFLS-C: green; LFLS-PM: green, hatched; HFHS-C: blue; HFHS-PM: blue, hatched. Data are represented as means ± s.e.m for panels **e**–**g** and median, interquartile range, minimum and maximum for panels **h**–**m**. Statistical analyses were performed using a two-way ANOVA followed by a Tukey post-hoc test. *P*-values of general effect for diet (D) and protein (P) factors and diet × protein (D × P) interaction are recorded under the title of each graph. Detailed significant differences detected by post-hoc test are recorded as follows: *p < 0.05, **p < 0.01, ***p < 0.001. Exact *p*-values for trends (0.05 ≤ *p*-value < 0.10) are recorded on graphs for an additional indication. See also Supplementary Figs. 3 and 4.

Importantly, the PM induced a significant shift in gut bacteria in both LFLS-fed and HFHS-fed mice that was independent of the effect of dietary fat and carbohydrate. Indeed, *Akkermansia muciniphila* was decreased by PM in both diets (Fig. 3a–e) and bacterial alpha-diversity displayed by Shannon and Simpson’s reciprocal indexes (Fig. 3f, g) were influenced most by protein sources. Accordingly, we also observed greater metabolic endotoxemia in PM-fed mice compared to their lean control, as revealed by the HFHS diet-induced increase in plasma LPS, which was significantly increased (*p* < 0.001) in HFHS-PM compared their LFLS-PM fed counterparts while only a tendency (*p* = 0.06) was observed between the HFHS-C and LFLS-C fed mice (Supplementary Fig. 4e). While histidine levels did not differ between HFHS-fed animals consuming casein *versus* PM, we felt it was still important to determine the circulating level of imidazole propionate (ImP), a microbially produced metabolite derived from histidine, since it was recently shown to impair insulin signaling via activation of mTORC1^18^. Neither plasma ImP nor its precursor urocanate was found to be affected by the protein source (Supplementary Fig. 4f, g). Irrespective of LFLS or HFHS consumption, the relative abundance of *Adlercreutzia*, *Tyzzerella,* and *Intestinimonas* genera were enriched while *Bacteroides* and the *Akkermansia* genera were depleted by the PM diet (Fig. 3a–d). Notably, *Tyzzerella*, *Adlercreutzia*, *Acetatifactor*, *Lachnospiraceae-UCG_006,* and *Ruminiclostrium_g* genera stood out as they were more abundant in both the HFHS context and in PM fed animals (Fig. 3b, d, and Supplementary Fig. 4a–d). This could suggest a potential association between the fecal enrichment of these bacteria and the exacerbation of the deleterious effects due to the HFHS and PM interaction.

Propionate, acetate, and butyrate are the main short-chain fatty acids (SCFA) produced in the gut and are mostly associated with a positive metabolic phenotype^19,20^ although this has been the subject of some debate^21^. SCFA are primarily derived from bacterial fermentation of fibers but can also be produced by protein fermentation^22^. Branched-chain fatty acids (BCFA) (e.g., isobutyric acid and isovaleric acid) are a class of SCFA produced in the gut upon proteolytic fermentation of branched-chain amino acids (BCAA). In the 12-week study, the fecal content of the major SCFA (acetic, butyric, and propionic acid) was all increased by the PM source in mice fed the LFLS diet. However, this protein effect was blunted in HFHS-fed animals (Fig. 3h–j). On the other hand, the minor SCFA (valeric, isobutyric, and isovaleric acid) were clearly increased by the PM source, and this effect was most pronounced in HFHS-fed animals (Fig. 3k–m).

### Early effects of the PM on gut microbiota, BCFA production and hepatic metabolism

**Fig. 4:**
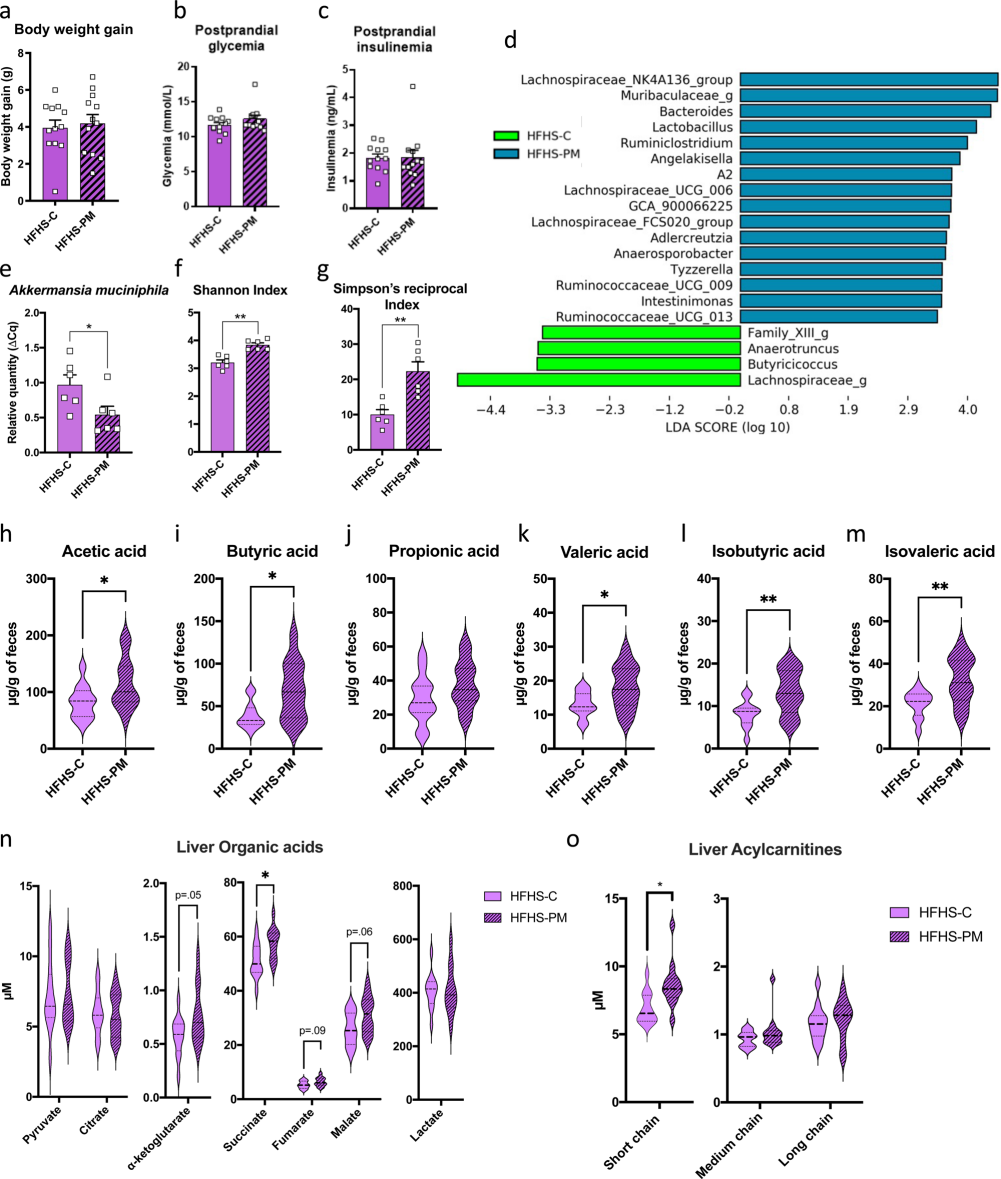
Two weeks of protein mix is not enough to affect weight gain and development of insulin resistance in HFHS-fed mice but induces changes in microbial composition, SCFA production, and postprandial metabolite profile. **a** Total body weight gain of mice fed with HFHS-C (purple) or HFHS-PM (purple, hatched) diet during a 2-week study. Mice were food-deprived for 12 h after 2 weeks of dietary treatment and food was given back to animals for 15 min. Blood was collected 30 min later by saphenous vein for postprandial (**b**) glycemia and (**c**) insulinemia assessment. (*n* = 12 biologically independent mice for both groups). Gut microbiota differences are represented by (**d**) a histogram plotting differentially abundant bacteria detected by LEfSe using a threshold of LDA score >2.5 between HFHS-C (green) and HFHS-PM (blue), (**e**) quantification of fecal *Akkermansia muciniphila* evaluated by gene expression through qPCR, and (**f**) Shannon and (**g**) Simpson’s reciprocal diversity indexes. *n* = 6 biologically independent mice for both groups. Mice selected for gut microbiota composition analysis were the closest to the median on main physiological parameters (body weight, glycemia, and insulinemia). Fecal levels of major SCFA (**h**) acetic, (**i**) butyric, and (**j**) propionic acid and minor SCFA (**k**) valeric, (**l**) isobutyric, and (**m**) isovaleric acid after 2 weeks of dietary intervention. Two hours following refeeding after the food deprivation, submandibular blood was collected for plasma metabolite profile determination. Mice were euthanized and muscle and liver were harvested for metabolite profiles. Hepatic (**n**) organic acid profile and (**o**) acylcarnitines (AC) profiles. *n* = 12 biologically independent mice for both groups. Data are represented as means ± s.e.m for panels **a**–**g** and median, interquartile range, minimum and maximum for panels **h**–**o**. Statistical analyses were performed using a two-tailed Student’s *t-*test or its nonparametric equivalent Mann-Whitney test. Detailed significant differences are recorded as follows: *p < 0.05, **p < 0.01. Exact *p*-values for trends (0.05 ≤ *p*-value < 0.10) are recorded on graphs for an additional indication. See also Supplementary Figs. 5–7 and Supplementary Tables 4–6. Short-chain AC: C2 to C4; Medium-chain AC: C5 to C10; Long-chain AC: C12 to C22.

To better understand the chronology of metabolic events and the mechanism linking our diet with diversified protein mix with worsening metabolic features, a short-term two-week study was next performed in mice fed either the HFHS-C or the HFHS-PM diet. At this early time point, there was no effect of the diet on body weight, food intake, or tissue composition (Fig. 4a and Supplementary Fig. 5a–h). Postprandial blood samples were collected to assess circulating glucose, insulin, and C-peptide levels, which were also found to be similar between the two groups (Fig. 4b, c, and Supplementary Fig. 5i). Immunoblotting also showed no changes in phosphorylation levels of S6, Akt, or IRS1 in the liver or skeletal muscle (Supplementary Fig. 4j–m).

However, at this early time point, there were clear differences in the microbiota from HFHS-C and HFHS-PM-fed mice (Fig. 4d). Of the 30 genera found in the 12-week protocol and the 40 genera found in the 2-week protocol, 27 were commonly detected, demonstrating reproducibility between both studies despite the change in treatment duration (Supplementary Figs. 3b, 6a). Thus, only two weeks of PM treatment was sufficient to induce gut microbiota changes similar to that observed after 12 weeks, such as enhanced alpha-diversity, modulated beta-diversity, and blooming of *Ruminiclostridium*, *Adlercreutzia,* and *Tyzzerella* bacteria with a decrease of the relative abundance of *Akkermansia* (a trend with LEfSe analysis—*p* = 0.05—confirmed by qPCR analysis) (Fig. 4d–g). This indicates that the changes in gut microbiota composition precede the metabolic impact of the mixed protein sources.

Importantly, we also observed that the fecal levels of SCFA and especially BCFA were already increased after only 2 weeks of the HFHS-PM diet, consistent with the 12-week study, demonstrating that the protein effect on these microbial metabolites took place prior to the development of obesity and insulin resistance (Fig. 4h–m).

Whereas this short-term diet intervention was not sufficient to perturb body weight gain, glucose homeostasis, and insulin action, changes in circulating and tissue metabolites were already present. In line with the higher glycine and lower proline content of the PM diet (Fig. 1b and Supplementary Table 3), we observed clear differences in the abundances of glycine across the plasma, liver, and skeletal muscle pools and lower levels of proline in plasma and skeletal muscle (Supplementary Fig. 7a–c). In the liver, we also observed an increase in glutamate/glutamine, aspartate/asparagine, and the urea cycle intermediate ornithine, a trend for higher levels of the tricarboxylic acid (TCA) cycle intermediates fumarate, malate, and alpha-ketoglutarate (αKG), as well as a significant increase in succinate (Fig. 4n and Supplementary Fig. 7b). These effects are likely due to the higher content of alanine and aspartate in the PM diet (Fig. 1b and Supplementary Table 3). Indeed, alanine and aspartate are both metabolized in the liver by the alanine aminotransferase (ALT) and aspartate transaminase (AST) enzymes, respectively, to yield glutamate which can feed both the urea and TCA cycles^23^.

In addition to the anticipated changes in amino acids, we observed a clear increase in the accumulation of even chain acylcarnitines in both plasma and liver but not the skeletal muscle of PM-fed mice (Fig. 4o and Supplementary Tables 4–6). Accumulation of even chain acylcarnitines results from mitochondrial overload and incomplete oxidation of fatty acids and is a hallmark of metabolic dysfunction^24^. Thus, our data demonstrate that the PM diet exerts an early effect on hepatic fatty acid metabolism prior to the onset of obesity or changes in insulin sensitivity.

### Transplantation of the fecal microbiota from HFHS-PM fed donor mice recapitulates some of the metabolic phenotypes in germ-free recipient mice

**Fig. 5:**
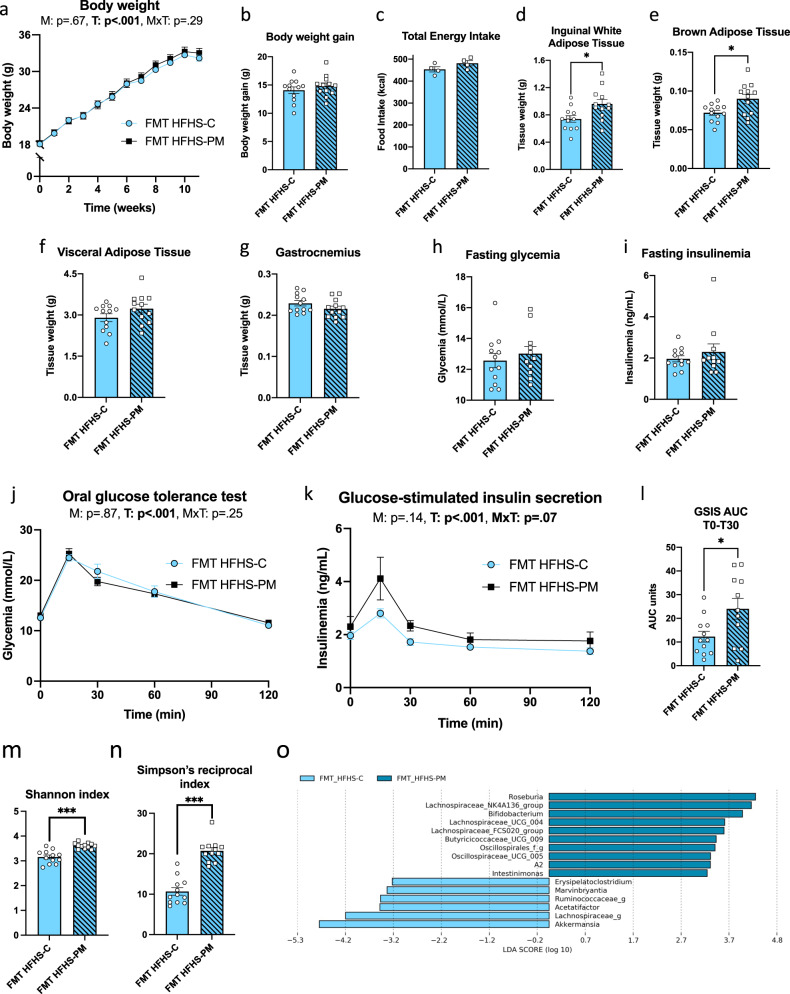
FMT is sufficient to induce some phenotypical changes caused by PM in a HFHS context. Germ-free mice were colonized by fecal slurry from either HFHS-C-fed mice (light blue) or HFHS-PM-fed mice (light blue, hatched). **a** Body weight curve, (**b**) total body weight gain, and (**c**) total energy intake of mice all fed HFHS-C and housed in an axenic-gnotobiotic facility. **d**–**g** Tissue weights of (**d**) inguinal white adipose tissue (iWAT) (**e**) intrascapular brown adipose tissue (BAT), (**f**) visceral adipose tissue (VAT), and (**g**) gastrocnemius muscle after 11 weeks. At week 10, mice were fasted for 6 h and challenged with an oral glucose load (1 mg/g body weight). **h** Glycemia and (**i**) insulinemia before the ingestion of glucose. **j** Glycemic response, (**k**) insulinemic response, and (**l**) area under the curve (AUC) for GSIS during the peak of insulin response (T0-T30), following the glucose load. Fecal microbiota alpha diversity represented by (**m**) Shannon and (**n**) Simpson’s reciprocal indexes. **o** Histograms of LDA scores identifying genera differentially represented between FMT HFHS-C (light blue) and FMT HFHS-PM (blue) groups, with a cut-off value of 2.5 for LDA score. *n* = 12 biologically independent mice for both groups except for panels **h**–**l** where *n* = 11 for FMT HFHS-PM group, and panel C where *n* = 4. Data are represented as means ± s.e.m. Statistical analyses were performed using a two-tailed Student’s *t-*test or its nonparametric equivalent Mann-Whitney test, or a two-way repeated measure ANOVA followed by a Tukey post-hoc test. *P*-values of general effect for microbiota (M) and time (T) factors and microbiota × time (M × T) interaction are recorded under the title of each graph. Detailed significant differences are recorded as follows: *p < 0.05, ***p < 0.001.

The interaction between diet, host, and gut microbiome is complex and thus we next evaluated the contribution of the gut microbiota to the metabolic phenotypes induced by the HFHS-PM diet by performing a fecal microbiota transplantation (FMT) study. Germ-free (GF) mice were colonized with fecal slurry from either HFHS-C or the HFHS-PM donor mice, which were then fed HFHS-C diet and maintained in our axenic-gnotobiotic animal facility to avoid interference from confounding environmental factors. While no significant effect was observed on body weight gain of the recipient GF mice, we found that the microbiota from the HFHS-PM donor animals increased the weight of adipose tissues (significant in inguinal and brown fat depots) as compared to GF mice colonized with the HFHS-C microbiota (Fig. 5a–g). Furthermore, whereas both groups exhibited similar fasting glucose and insulin, and glucose excursions during a GTT were comparable (Fig. 5h–j), mice bearing the microbiota from HFHS-PM donors showed an exacerbated glucose-stimulated insulin response (Fig. 5k) as compared to their counterpart mice bearing the fecal microbiota from HFHS-C donors, which was highly significant (post-hoc analysis: *p* = 0.006) at the 15 min peak of insulin secretion), and further illustrated by calculation of the GSIS during the peak of insulin response (Fig. 5l). 16S rRNA gene sequencing of the gut microbiota composition revealed that FMT-HFHS-PM mice had greater alpha diversity than FMT-HFHS-C mice, represented by Shannon and Simpson’s reciprocal indices, consistent with data from donor mice (Fig. 3f, g and 5m–n). The recipient mice also exhibited similar changes in their bacterial taxa composition as seen in their donor counterparts, as reflected by an overrepresentation of *Akkermansia muciniphila* in mice transplanted with the HFHS-C fecal samples and an overrepresentation of a genus from the *Lachnospiraceae*_NK4A136_group, *A2,* and *Intestinimonas* in the mice transplanted with HFHS-PM fecal samples (Figs. 3b, d and 5o). Thus, major taxonomic changes in donor mice were successfully transferred by FMT even if all recipient mice were fed the same diet. These data demonstrate that changes in the composition of the gut microbiota are involved in at least part of the phenotypic effects of the HFHS-PM diet, notably contributing to the greater body fat accretion and insulin resistance as demonstrated by the higher insulin secretion required to achieve glucose control.

### Intact gut microbiota is required for BCFA production in HFHS-PM fed mice

**Fig. 6:**
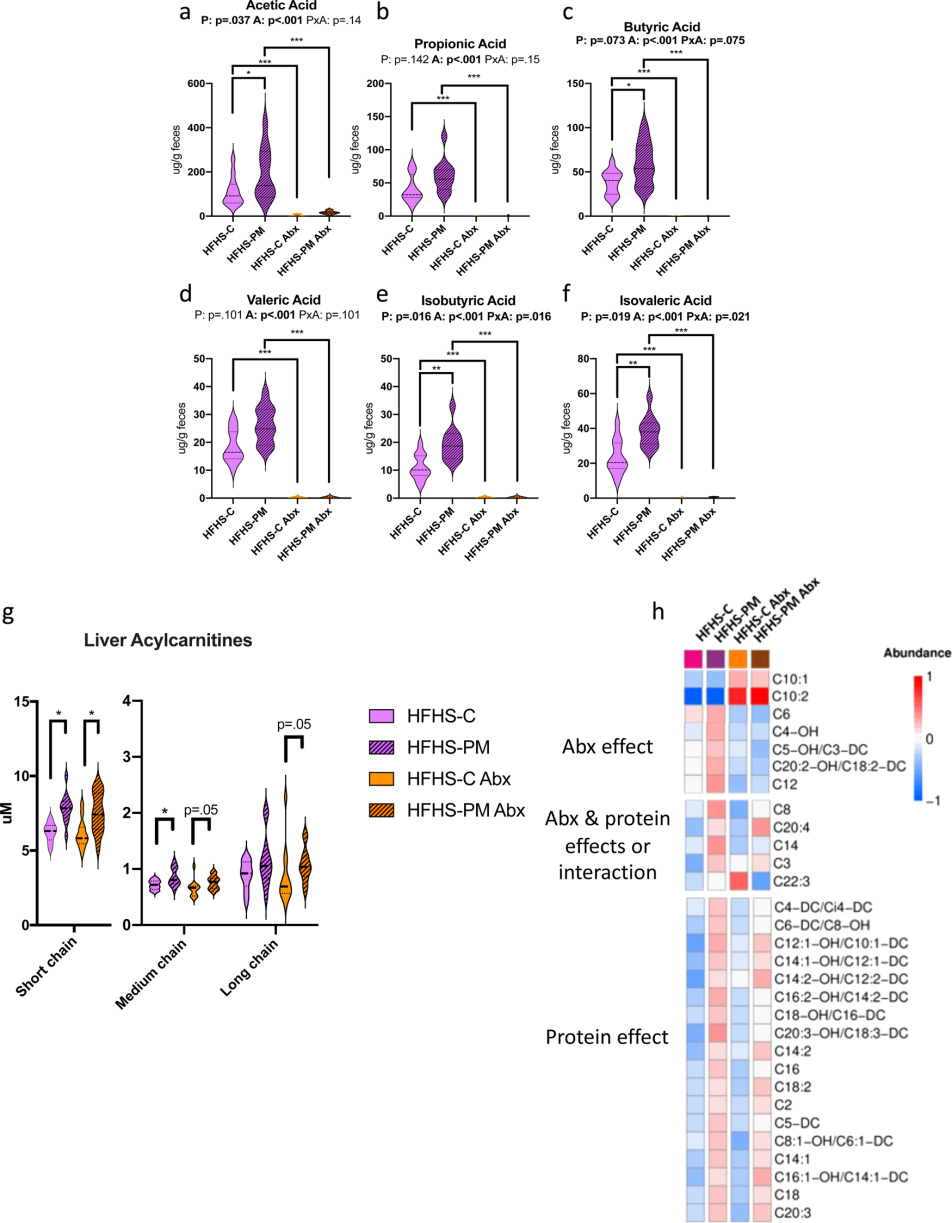
Antibiotics (Abx) prevent SCFA and BCFA production while mitigating diet impact on hepatic acylcarnitines. Mice were fed with a HFHS-C (purple) or a HFHS-PM (purple, hatched) and administered with an antibiotic cocktail (orange, and orange, hatched, respectively). Fecal levels of major SCFA (**a**) acetic, (**b**) propionic, and (**c**) butyric acid, and minor SCFA (**d**) valeric, (**e**) isobutyric, and (**f**) isovaleric acid after 2 weeks of dietary intervention. Hepatic (**g**) acylcarnitine profile and specific (**h**) acylcarnitine changes attributed to antibiotic treatment, protein source, or both. Short-chain AC: C2 to C4; Medium-chain AC: C5 to C10; Long-chain AC: C12 to C22. Data are represented as median, interquartile range, minimum and maximum. Metabolite concentrations were log10 transformed and Pareto scaled before generating a heatmap. Statistical analyses were performed using a two-way ANOVA followed by a Tukey post-hoc test. *n* = 12 biologically independent mice for all groups. *P*-values of general effect for protein (P) and antibiotic (A) factors and protein × antibiotic (P × A) interaction are recorded under the title of each graph. Detailed significant differences detected by post-hoc test are recorded as follows: *p < 0.05, **p < 0.01, ***p < 0.001. Exact *p*-values for trends (0.05 ≤ *p*-value < 0.10) are recorded on graphs for an additional indication. See also Supplementary Figs. 7 and 8.

To gain further insight into the role of the gut microbiota in the metabolic consequences of PM feeding, we carried out a second 2-week study with additional groups of mice receiving broad-spectrum antibiotics to disrupt the microbiota. As expected from previous work^25^, antibiotic treatment alone also had some impact on postprandial glycemia and insulinemia (Supplementary Fig. 8a, b), as well as body and tissue weights, (Supplementary Fig 8c–i), which did not seem to be related to energy intake (Supplementary Fig. 8j). Nonetheless, antibiotic-mediated disruption of the microbiota markedly blunted the HFHS-PM-induced fecal production of SCFA and BCFA (Fig. 6a–f) confirming that the microbiota was responsible for their raised levels upon dietary treatments. We also fully reproduced the early and diet-dependent changes in hepatic acylcarnitines (AC) from the previous 2-week study (Fig. 6g–h, and Supplementary Table 7). Interestingly, whereas antibiotic treatment completely abolished the effect of PM-feeding on SCFA and BCFA, only a small fraction of the PM-diet effects on hepatic AC were altered by antibiotic treatment. Notable, among these 3-hydroxyisovaleryl (C5-OH) and 3-hydroxyisobutyryl (C4-OH) which were raised by PM-feeding but blunted with antibiotic treatment, are produced from mitochondrial oxidation of the BCFA, isovaleric, and isobutyric acid, respectively^11,26^. These results highlight the selective role of the gut microbiota in the regulation of these key metabolites and demonstrate that not all metabolic effects of PM-feeding are driven by the changes in the gut microbiota.

### BCFA elevate glucose production and activate mTORC1/S6K1 signaling in hepatocytes

**Fig. 7:**
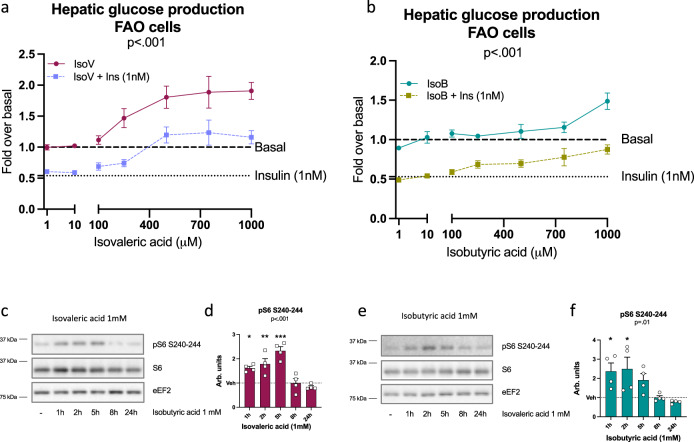
BCFA increases hepatic glucose production and activates the mTORC1/S6K1 pathway in vitro. Branched-chain fatty acids (BCFA) effect on hepatic glucose production in hepatocytes (FAO) in basal or insulin (1 nM) condition with (**a**) isovaleric (isoV) or (**b**) isobutyric (IsoB) acid (1–1000 µM); data corrected for total protein. For IsoV treatment in basal condition (dark red): *n* = 6 independent experiments for 1 µM, 500 µM and 750 µM, *n* = 7 for 10 µM and 250 µM, *n* = 11 for 1 mM, and *n* = 13 for 100 nM. For IsoV treatment in insulin condition (light purple): *n* = 5 independent experiments for 750 µM, *n* = 6 for 1 µM, *n* = 7 for 10 µM, *n* = 8 for 250 µM and 500 µM and *n* = 12 for 100 µM and 1 mM. For IsoB treatment in basal condition (blue-green): *n* = 6 independent experiments for 250 µM, *n* = 7 for 1 µM, 10 µM, 500 µM and 750 µM, *n* = 13 for 100 µM, *n* = 15 for 1 mM. For IsoB treatment in insulin condition (khaki green): *n* = 6 independent experiments for 750 µM, *n* = 7 for 1 µM, 10 µM and 250 µM, *n* = 9 for 500 µM and *n* = 14 for 100 µM and 1 mM. Immunoblots and quantification of densitometry analyses for pS6 S240-244 and total S6 in FAO cells exposed to 1 mM of (**c, d**) isovaleric (dark red) and (**e, f**) isobutyric (blue-green) acid. eEF2 has been used as a loading control and *n* = 4 independent experiments. Data are means ± s.e.m. A Kruskal-Wallis test followed by a two-tailed Dunn’s post-hoc test versus Vehicle/Vehicle + Insulin was performed in each condition and a general *p*-value is recorded under the title of each graph. Detailed significant differences detected by post-hoc test are recorded as follows: *p < 0.05, **p < 0.01, ***p < 0.001. See also Supplementary Fig. 9.

In the animal studies, changes in isobutyric and isovaleric acid were the most striking as their content was not only driven by HFHS diet but also by the PM and by their interaction (Fig. 3l–m). Moreover, the production of these BCFA and that of their hepatic oxidation intermediates was completely blunted with antibiotic treatment in HFHS-PM diet-fed mice. While they have been reported to be increased in obesity^27^ and in non-alcoholic fatty liver disease (NAFLD)^28^ the impact of these BCFA on host metabolism has not been directly studied. Here, we evaluated their impact on glucose metabolism in hepatic and muscle cells. We found that both isobutyric acid and isovaleric acid dose-dependently increased hepatic glucose production (HGP) in FAO hepatoma cells (Fig. 7a–b). Isovaleric acid was more potent as its glucose-producing action was detectable at 250 µM whereas the effect of isobutyric acid was only observed at 1 mM. BCFA increased glucose production even in the presence of insulin, but isovaleric acid was again more potent than isobutyric acid to overcome the suppressive effect of insulin. This impact on hepatic glucose metabolism was associated with robust but transient activation of the mTORC1/S6K1 pathway in these cells as revealed by increased S6 phosphorylation (Fig. 7c–f). On the other hand, L6 myotubes treated with BCFA displayed no change in glucose uptake (Supplementary Fig. 9a, b). These in vitro findings point towards the liver as the main target of BCFA, which aligns with the mTORC1/S6K1 and insulin signaling data from our in vivo observations.

## Discussion

The role of dietary lipids and carbohydrates in the development of obesity and associated cardiometabolic diseases has been a major focus over the last 50 years. However, the potential role of dietary proteins in these pathologies remains poorly studied. We show here that inclusion of a diversified mix of proteins mirroring the western diet in a HFHS regimen causes early broad changes in the gut microbiota, selectively increases the production of BCFA, and induces early alterations in hepatic lipid oxidation which promote hepatic mTORC1/S6K1 activation, liver insulin resistance, and elevated gluconeogenesis. Importantly, these changes in systemic insulin levels and hepatic lipid oxidation were observed upon altering the source of dietary protein without modifying total protein intake and were coupled with impaired BAT thermogenesis, leading to higher lipid deposition in adipose tissues and exacerbating HFHS-induced obesity.

Central to this concept is the early and robust effects of the PM diet on gut microbial populations. Remarkably, the PM significantly reshaped the gut microbial communities within two weeks of the commencement of the PM diet. Since the shift in gut microbiota occurred prior to any change in systemic insulin sensitivity or body weight gain we interpret this to mean that the microbial changes underlie at least part of the obesogenic and insulin desensitizing effects of PM inclusion in the HFHS diet. This is further supported by the FMT studies in GF mice where we have shown the direct contribution of the gut microbiota by showing that fecal slurry from the HFHS-PM fed donor mice could reproduce some key metabolic phenotypes such as adipose tissue accretion and insulin resistance as shown by the exacerbated insulin responses during the OGTT. Further studies will be needed to disentangle the individual versus the combined role of the microbiome versus the diet per se in recapitulating the effect of HFHS-PM treatment on other key phenotypes such as increased body weight and aberrant liver function.

Indeed, the gut ecosystem has emerged as a central hub for mammalian energy intake regulation by processing nutrients into absorbable bioactive compounds^29^. Bacterial populations are modulated by diet and, in return, can produce metabolites that can either be used as fuel for microbial cross-feeding or pass into the host circulation and exert beneficial or harmful effects^30^. The fermentable fibers constitute the major energy source for bacteria, but their availability declines along the intestine^31^. Thus, peptides and proteins are degraded in the distal colonic populations, leading to the production of a broad spectrum of metabolites^30^ such as BCFA like isobutyric and isovaleric acid which is derived from valine and leucine, respectively^32–34^.

Unlike the well-studied major SCFA, (acetic, butyric, and propionic acid) data on the metabolic effects of BCFA is sparse. Yet, metabolomic studies in fecal human samples show that isovaleric acid is increased in obese patients compared to lean ones^27^, that isobutyric acid is higher in NAFLD patients compared to healthy controls^28^ and that both isobutyric acid and isovaleric acid are increased in patients with hypercholesterolemia^35^. These data suggest that BCFA might play a pathologic role in cardiometabolic disease. In our study, we showed that fecal excretion of BCFA increased with consumption of HFHS diet compared to LFLS diet, and this effect was greatest with PM consumption compared to casein. Our in vitro studies further revealed the gluconeogenic potential of isobutyric and isovaleric acids and their ability to activate the mTORC1/S6K1 pathway, highlighting a novel gut-liver cross-talk that appears to underlie the deleterious interaction of HFHS diet and PM. These results are consistent with Hu et al. who identified 3-hydroxyisobutyrate (3-HIB), which is derived from valine and isobutyric acid^36^, as a substrate for gluconeogenesis^37^. It was also shown that 3-HIB can stimulate endothelial fatty acid uptake in muscle and is associated with T2D^38^. Another in vitro study demonstrated that isobutyric and isovaleric acid had the ability to block lipolysis in rat and human adipocytes^39^. The authors of this study suggested that this effect of BCFA might promote insulin sensitivity, however, an alternate outcome could be greater adipose tissue expansion as was observed with HFHS-PM-fed mice in our study.

Among the many effects of PM on the gut microbiota, we found that PM particularly depleted *Akkermansia muciniphila* which occurred after only 2 weeks of dietary exposure and preceded any changes in body weight gain or glucose homeostasis. This effect was also found in colonized GF mice after FMT from HFHS-PM fed mice as compared to GF mice after FMT from HFHS-C fed mice. The high abundance of this bacterium in HFHS-fed mice has been associated with protection against obesity and an increase in insulin sensitivity^40^. Moreover, its administration as a probiotic notably improved metabolic health in both obese mice and humans^41–43^. Our data indicate that casein promotes *Akkermansia muciniphila* and thus could have a protective effect on the development of obesity when used in obesogenic murine diets. On the contrary, the PM favored a more diversified gut microbiota composition, while disturbing the ecological niche of *Akkermansia muciniphila*. In addition, the HFHS-PM interaction also promoted enrichment of *Tyzzerella* that was previously correlated with the lifetime risk of cardiovascular diseases^44^ and a low dietary quality^45^.

Other than early changes in microbial populations and microbe-derived SCFA, we also observed robust acylcarnitine accumulation in the liver and plasma of HFHS-PM-fed mice. Compared to casein, PM induced an overall increase of short, medium, and long-chain acylcarnitines in the plasma. Elevated circulating even chain acylcarnitines (particularly short and long-chain) is a hallmark of metabolic disease, present in T2D patients^46^, and may be a predictive marker of prediabetes^47^. However, it is unclear if they promote insulin resistance or simply represent biomarkers of dysregulated metabolism^48^. The acylcarnitine profile in HFHS-PM-fed animals and the increase in multiple TCA intermediates observed in the liver is consistent with a state of mitochondria overload and suggests the combination of PM with a HFHS diet interferes with hepatic lipid oxidation. It is interesting that abrogation of gut microbiota by antibiotic treatment did little to blunt these broad changes in specific hepatic acylcarnitines, suggesting that not all the metabolic effects of PM-feeding can be attributed to changes in the microbiota. These effects are reminiscent of those observed with modulation of dietary BCAA supply^26,49^ and suggest that altered amino acid supply might account for this effect of the PM diet. Thus, additional studies are warranted to determine which component of the PM diet is responsible for the rapid shift in hepatic acylcarnitine metabolism.

The role of the mTOR signaling pathway in obesity and energy metabolism has been thoroughly investigated in the last two decades. This nutrient-sensing pathway allows cells to react to environmental factors in insulin target tissues, such as muscle, liver, and fat^50^ and can be activated by different intracellular and extracellular signals. It has been shown that mTOR can be activated by amino acids, particularly by BCAA^11^. However, we did not detect any difference in BCAA levels in circulation nor in tissues in PM-fed mice, rather our studies suggest that BCFA are responsible for the increased mTORC1/S6K1 activation observed in the liver of mice fed the protein mix. Koh et al. recently demonstrated that microbially-produced ImP is a key metabolite increased in type 2 diabetic patients that can activate the mTORC1 pathway^18^. However, we have measured ImP in this study and found no differences between mice fed casein and the PM sources indicating that this microbial product is not contributing to the effect of the mixed dietary proteins on hepatic mTORC1/S6K1 activation and insulin resistance. Another potential mechanism of PM-induced hepatic insulin resistance is the incomplete oxidation of lipids by the TCA cycle which can lead to the accumulation of other lipid intermediates such as DAG which activate stress-sensitive kinases, such as PKC that can negatively phosphorylate IRS to promote insulin resistance. This potential mechanism is supported by our observations of increased levels of acylcarnitines and protein levels of PKC theta, a well-known IRS-1 S1101 kinase, that was induced by the combination of HFHS and PM.

Our findings are relevant to the use of pre-clinical models in metabolic investigations. We demonstrate that altering the source of dietary protein without modifying total protein intake has a major impact on the microbial and metabolic effects of obesogenic diets and should be considered when designing dietary studies in animal models of obesity and diabetes. Notably, we found that the inclusion of a protein mixture that mirrors the protein composition of the western diet accentuates the effects of HFHS feeding on weight gain, hepatic insulin sensitivity, and gut microbiota composition compared to the classical casein-based diet that is still widely used. In this regard, while we tried our best to match the HFHS-C and HFHS-PM diets as much as possible (including matching for SAT:PUFA ratio), we cannot completely exclude the potential contribution of other minor nutritional factors we could not completely adjust for. This includes monounsaturated fatty acids, some fibers that we could not discriminate according to the soluble/insoluble classification, as well as potential advanced glycation end-products issued from meat cooking. However, since these non-adjusted components represent less than 5% of the energy intake variation between the diets used, their impact is most likely negligible as compared to that conferred by the major differences in protein composition.

In summary, we have demonstrated that dietary protein sources have a major impact on obesity and insulin resistance that is mechanistically linked to direct and early changes in gut microbiota and BCFA production, leading to activation of the mTORC1/S6K1 pathway and hepatic insulin resistance. Cellular studies further provided direct evidence that BCFA can exert cell-autonomous effects on mTORC1/S6K1 activation and stimulate hepatic gluconeogenesis. Our work thus reveals the importance of considering the dietary protein source when designing nutritional interventions aimed at improving weight management, and that modifying the composition of dietary proteins may offer therapeutic benefits for insulin resistance and T2D.

## Methods

### Diet

For this study, we have elaborated low-fat low sucrose (LFLS: 75% kcal carbohydrates, 10% kcal sucrose, 10% kcal fat) and high-fat high-sucrose (HFHS: 35% kcal carbohydrates, 30% kcal sucrose, 50% kcal fat) homemade diets containing a protein mix (PM) designed to represent human consumption according to the US Department of Agriculture data^51^. The corresponding LFLS and HFHS diets containing casein (C) as the only protein source were used as controls. The protein sources consisted of cooked meat (chicken/pork/beef, Happy Yak, Canada), fish (cod, Seagarden, Norway), plant (soy, Teklad Envigo, USA and pea/rice, Canadian Protein, Canada), egg (egg white, Teklad Envigo, Canada) and dairy (whey, Canadian Protein, Canada and casein, MP Biomedicals, USA). All of the protein sources were provided in a lyophilized form, and details of their process are available on the respective websites of the providers, with the exception of beef, pork, and chicken (Happy Yak, Canada), which are lyophilized lean cuts of meat without any additional processing. The composition of each protein source used in the PM has been analyzed (Environex) and the information is available in Supplementary Table 3. The resulted PM consisted of 81% protein, 11% fat, 3% carbohydrates, 1% fiber, and a negligible amount of ash and humidity. Diets were then matched for protein, fat, carbohydrate, fiber. All diets were isonitrogenous (15% kcal) and lard and corn oil content were adjusted such that the saturated: polyunsaturated fatty acid ratio (SAT: PUFA) in the four experimental diets represented the daily American dietary intake^9^ (Supplementary Tables 1 and 2).

### Animals

Animals were single-housed, except for the GF study, where mice were housed 3 per cage, all animals were housed in ventilated cages at a humidity of 40–50% and a temperature of 22 °C on a 12-h dark-light cycle with ad libitum food. 6-week-old male C57BL/6J mice for the 12-week protocol,10-week-old male C57BL/6J mice for the 2-week protocol, and 11-week-old male C57BL/6J mice for the 2-week antibiotic protocol (The Jackson Laboratory, USA) were acclimatized for 2 weeks on the LFLS-C diet prior to the beginning of their respective treatment. Animals were then distributed into treatment groups accordingly to their body weight and fed with their respective diets for 12 weeks (LFLS-C, LFLS-PM, HFHS-C, or HFHS-PM) or 2 weeks (HFHS-C or HFHS-PM). Food intake was measured three times a week and bodyweight weekly. Body composition was measured by nuclear magnetic resonance with a Bruker Minispec (LF90) apparatus. Fresh feces were collected to analyze gut microbiota composition, as well as short-chain and branched-chain fatty acids content.

For the 12-week protocol, a fasting blood sample was collected at week 5 and an oral glucose tolerance test, with a dose of 1 mg of glucose/g of body weight, was performed at week 11, both after a 6-h fast. Sacrifice was done at week 12 after a 6-h fast and animals were injected intravenously with saline or insulin (3.8 U/kg) 5 min prior to sacrifice for analysis of insulin signaling in the tissues. For the 2-week antibiotic protocol, half the mice had antibiotics (1 g/L of Ampicillin and 0.5 g/L of Neomycin), in their drinking water changed 3 times per week in bottles wrapped in aluminum foil, one week prior to the start or the HFHS diets and for the whole duration of dietary intervention. For both 2-week protocols, a meal-test was conducted at the end of the protocol, where 0.5 g of their respective diet was given to the animals after a 12-h fast. The mice had 15 min to eat the food and were euthanized 2 h after the end of the meal test.

For the fecal microbiota transplantation (FMT) study, 8–9-week-old male C57BL/6 J germ-free mice (International Microbiome Center, University of Calgary) were ordered and colonized upon arrival in the axenic-gnotobiotic sector of our animal facility. For fecal microbiota transplantation solutions, fecal samples from each of the 15 mice from groups HFHS-C and HFHS-PM of the first 12-week protocol were, respectively, pooled and diluted in sterile cold PBS at 2 ml/100 mg of feces, vortexed 3 min, and centrifuged 3 min at 800 × *g* at 4 C. Stool suspension was then aliquoted and thawed only once, when used for fecal microbiome transplant, where each mouse received 200 μl of the solution by gavage. Upon arrival, mice were separated into two groups according to BW (FMT-HFHS-C of FMT-HFHS-PM) and received the first colonization. Every 2 weeks for the whole duration of the study mice received another FMT. Mice were group-housed 3 by 3 on a 12-h dark-light cycle with ad libitum food. Food intake was measured three times per week, while body weight was assessed weekly. Animals were thoroughly observed for any signs of fighting within each cage, and none were noted. All mice were fed HFHS-C diet.

All animals were euthanatized by cardiac puncture (under isoflurane anesthesia) and cervical dislocation before tissue collection for analysis. All manipulations were approved (#2017-156-1) by the *Comité de protection des animaux de l’Université Laval* (CPAUL) and complied with the Canadian Council on Animal Care (CCAC) guidelines.

### Biochemical analyses

ELISA assays were used to measure insulin (Alpco Mouse Ultrasensitive Insulin kit), c-peptide (Crystal Chem Mouse C-Peptide kit) and lipopolysaccharides (LPS) (MyBioSource Mouse Lipopolysaccharides kit) in circulation according to manufacturer’s instructions. Imidazole propionate and urocanate were measured in plasma according to the method previously described^18^. Briefly, plasma samples were extracted with 10 volumes of acetonitrile containing deuterated internal standards. The supernatant was dried and the imidazole propionate and urocanate were derivatized into butyl esters using butanol: HCL (conc) [95:5]. The samples were separated on a BEH C18 column (Waters, Milford, USA) using a gradient consisting of water with 0.1% formic acid (A-phase) and acetonitrile with 0.1% formic acid (B-phase). Mass spectrometric analysis was performed using an TQ-XS mass spectrometer (Waters, Milford, USA). The imidazole propionate and urocanate were detected by multiple reaction monitoring using the transitions 197/81 and 195/93, respectively. Quantification was made using external standard curves. Amino acids, acyl-carnitines, and organic acids were measured using stable isotope dilution techniques. Amino acids and acyl-carnitine species were measured using flow injection tandem mass spectrometry, organic acids profile was determined by capillary gas chromatography-mass spectrometry (TRACE ISQ instrument, Thermo Election Corporation). Samples were equilibrated with a cocktail of internal standards, deproteinized by precipitation with methanol, aliquoted supernatants were dried, and then esterified with hot, acidic methanol (acyl-carnitines), or n-butanol (amino acids)^26^.

### Immunoblotting

Liver and gastrocnemius muscle were powdered with liquid nitrogen and then homogenized under rotation for 2-h at 4 °C in a 10-fold mass excess of ice-cold lysis buffer (50 mM Hepes pH 7.5, 150 mM NaCl, 1 mM EGTA, 20 mM β-glycerophosphate, 1% NP-40, 10 mM NaF, 2 mM Na_3_VO_4_, 0.1 mM PMSF and protease inhibitors cocktail). Lysates were clarified by centrifugation at 16,000 × *g* for 10 min at 4 °C and the proteins were measured with a BCA assay (ThermoFisher Scientific, Burlington, Canada). Tissue lysates (5–30 μg) were denatured in SDS sample buffer and submitted to SDS-PAGE followed by transfer on nitrocellulose membranes (Pall Corporation, Mississauga, Canada). Membranes were blocked for 1-h at room temperature and then probed with the primary antibody overnight at 4 °C. After washing in TBST (50 mM Tris-HCl pH 7.5, 0.15 mM NaCl, and 0.1% Tween-20), the membranes were incubated with HRP-conjugated secondary antibodies for 1-h at room temperature. Cells were washed with PBS and lysed in ice-cold lysis buffer (50 mM Hepes pH 7.5, 150 mM NaCl, 1 mM EGTA, 20 mM β-glycerophosphate, 1% NP-40, 10 mM NaF, 2 mM Na_3_VO_4_, 0.1 mM PMSF and protease inhibitors cocktail). Cell lysates were clarified by centrifugation at 16,000 × *g* for 10 min at 4 °C and the proteins were measured with a BCA assay (ThermoFisher Scientific, Burlington, Canada). Lysates in the SDS sample buffer were then subjected to immunoblotting. The detection was performed with an ECL reagent (Millipore, Etobicoke, Canada). See Supplementary Method Table 1 for the full details on the antibodies used and the immunoblotting conditions. Immunoblots were analyzed using ImageJ software (NIH, Bethesda, USA). Uncropped and unprocessed scans of blots from representative gels are found in the Data Source file.

### Quantitative PCR analysis

RNA was extracted from freeze-powdered brown adipose tissue with the Direct-zol RNA Miniprep Plus kit according to the manufacturer’s instructions (Zymo Research, Irvine, USA). Gene expression was assessed by the ΔΔCt method and *Actin* and *Hprt* were used as the reference genes. *Akkermansia muciniphila* bacterial quantification was performed from extracted fecal bacterial DNA. Primer sequences are available in Supplementary Method Table 2.

### 16S rRNA amplicon sequencing

Fecal DNA was extracted from fresh feces with the ZymoBIOMICS DNA Miniprep kit (Zymo Research) according to manufacturer’s instructions and then sent to the sequencing platform at *Institut de biologie intégrative et des systèmes* (IBIS) for PCR amplification of the V3-V4 region and 16S rRNA sequencing analysis. Forward and reverse primers were removed from 16S rRNA gene amplicons using Cutadapt (v3.1^52^). Sequence reads were analyzed using the DADA2 package (v1.145.0^53^) in R (v3.6.0; http://www.R-project.org). Forward and reverse reads were first trimmed at 270 bp and 205 bp, respectively, to remove low-quality regions. Sequences with an expected error threshold >2 and >4 for the forward and reverse reads, respectively, with ambiguous bases, and with a quality score, less than 3 or equal to 2 were discarded. Dereplication and denoising of filtered sequences were carried out using DADA2 default parameters. Denoised forward and reverse reads were merged (all reads with any mismatches were removed) and searched for chimeras. Taxonomic assignment of amplicon sequence variants (ASVs) was performed using the RDP classifier algorithm (v2.2^54^) trained against the Silva database 132^55^ (https://zenodo.org/record/1172783#.YGEfARKQikA). In order to normalize sampling effort, samples were rarefied to an even sampling depth of 10459 sequences. ASVs with a number of sequences <0.05% of total number of sequences and present in less than 3 samples were discarded at this step. A phylogenetic tree was built in R using the DECIPHER (v2.14.0^56^) and phangorn packages (v2.5.5^57^). Data visualization and analyses were performed in R with the phyloseq (v1.28^58^) package. Overall bacterial community composition was visualized in heatmaps at the genus level. The presence of “f” or “g” at the end of taxon denotes an unclassified family or genus, respectively. To quantify bacterial alpha diversity, Shannon and Simpson’s reciprocal indexes were calculated. Principal coordinates analysis (PCoA) was performed on an unweighted UniFrac distance matrix in order to measure beta diversity. The statistical significance of differentially abundant bacteria between the two distinct biological conditions was measured with LEfSe^59^. A *p*-value of <.05 and a linear discriminant analysis (LDA) score of ≥2.5 will be considered statistically significant. The raw sequence data generated in this work were deposited into the European Nucleotide Archive (ENA) under accession PRJEB37442.

### Short-chain fatty acids (SCFA)

SCFA were measured in the feces of all the animals at week 11 for the 12-week study and at week 2 for the 2-week studies. SCFA were extracted according to a protocol previously published by García Villalba et al.^60^ with some modifications. Right after collection, feces were weighed and 1 mL of 0.5% phosphoric acid was added per 100 mg of material. Fecal suspensions were homogenized 2 min with a Bead Ruptor 12 (Omni International, Kennesaw, GA, USA), then centrifuged at 18,000 × *g* for 10 min at 4 °C. The supernatant was collected and an equal volume of ethyl acetate, spiked with internal standard 4-methylvaleric acid were added. To extract SCFA, samples were mixed 2 min at 2400 rpm using a VWR VX‑2500 Multitube Vortexer (VWR, Radnor, PA, USA), then centrifuged at 18,000 × *g* for 5 min at 4 °C. The organic phase was transferred to an autosampler vial for for gas chromatography analysis. A 5-point calibration curve were prepared with a mix of acetic acid, propionic acid, butyric acid, isobutyric acid, valeric acid, isovaleric acid, and internal standard 4-methyl valeric acid. SCFA quantification was performed on a Shimadzu GC 2010 Plus equipped with a Nukol Supelco capillary GC column (30 m × 0.25 mm id, 0.25 µm) and a FID detector.

### Cell culture and stimulation

FAO rat hepatocytes cells were cultured in RPMI 1640 containing 10% FBS. L6 rat myoblasts (kind gift from Dr. Amira Klip, Hospital for Sick Children, Toronto, ON, Canada) were grown in α-MEM with 10% FBS and differentiated into myotubes in α-MEM with 2% FBS. For immunoblotting, FAO cells were serum-deprived for 24 h and treated with or without isobutyric acid (1 mM) (MilliporeSigma Canada, Oakville, ON, Canada) or isovaleric acid (1 mM) (MilliporeSigma Canada) for the last 1–24 h of deprivation.

### Glucose production

FAO cells were incubated 16–18 h in serum-free medium, with or without insulin (1 nM), isobutyric acid (1–1000 μM) (MilliporeSigma Canada, Oakville, ON, Canada), or isovaleric acid (1–1000 μM) (MilliporeSigma Canada). The cells were washed three times with PBS and then incubated with phenol-free and glucose-free DMEM medium supplemented with 20 mM sodium L-lactate and 2 mM sodium pyruvate for 5 h with or without the indicated concentration of insulin (1 nM), isobutyric acid (1–1000 μM) or isovaleric acid (1–1000 μM). Cell supernatants were collected, and glucose concentration was measured with the Amplex-Red Glucose assay kit (Invitrogen, Burlington, ON, Canada) according to the manufacturer’s instructions. Cells were lysed with 50 mM NaOH and protein concentration was determined using a BCA protein assay kit to normalize glucose production.

### Measurement of 2-deoxyglucose uptake

Fully differentiated L6 myotubes were serum-deprived for 5 h in α-MEM and treated with isobutyric acid (0.1–1000 μM) or isovaleric acid (0.1–1000 μM) for the last 2 h of deprivation. The cells were also stimulated with or without insulin (100 nM) during the last 30 min of deprivation. Glucose uptake was measured in cells incubated for 8 min in HEPES-buffered saline containing 10 μmol/l unlabeled 2-deoxyglucose and 0,33 µCi/ml 2-[1,2-3H(N)]-deoxy-D-glucose (Perkin Elmer). The reaction was terminated by washing three times with ice-cold 0.9% NaCl. Cell-associated radioactivity was determined by lysing the cells with 0.05 N NaOH, followed by liquid scintillation counting and normalization to protein concentration.

### Statistical analysis

Data are expressed as means ± s.e.m. For the 12-week and the antibiotic protocols, a two-way or a three-way analysis of variance (ANOVA) was performed to isolate the main effects of diet (D), protein (P) insulin condition (I), or antibiotic condition (A) factors, as well as the corresponding interaction effects. A Tukey post-hoc test with a type I error set at 0.05 was then applied (SigmaPlot® v12.0, San Jose, CA, USA) when the *p* < 0.10 threshold of main factors was achieved. The exact *p*-values for main effects are indicated under the title of each graph and subsequent significant differences established by post-hoc test were recorded as follows: *p < 0.05, **p < 0.01, ***p < 0.001. Trends (0.05 ≤ *p*-value < 0.10) were also reported in graphs for an additional indication. Data violating ANOVA normality and homoscedasticity postulates were log-transformed before the test was applied. Differences in parameters involving repeated measures were assessed using a mixed linear model (proc MIXED procedure) in SAS (SAS Studio, SAS® University Edition USA), with treatment, time (T) and treatment*time interaction as fixed effects and an autoagressive (1) covariance matrix to account for within-subject correlations. The skewness in the distribution of model residuals was considered and data were log-transformed when required. For the 2-week and the germ-free protocols a two-tailed Student’s *t* test or its non-parametric equivalent Mann-Whitney test when data normality and homoscedasticity were not respected was performed and significant differences were recorded as follows: *p < 0.05, **p < 0.01, ***p < 0.001 For BCFA measurement in vitro, a Kruskal-Wallis one-way ANOVA followed by a two-tailed Dunn’s post-hoc test versus basal or insulin was performed in each basal or insulin condition. *P*-values of general ANOVA are indicated under the title of each graph. Features were considered statistically different at *p* < 0.05 and significant differences were recorded as follows: *p < 0.05, **p < 0.01, ***p < 0.001 Trends (0.05 ≤ *p*-value < 0.10) are recorded on graphs for additional indication. Statistics regarding metataxonomic analysis are described in the corresponding subsection (see above). Microsoft Excel (v16.47.1) and Graphpad Prism (v9.1.0) software were used to compile data and make graphs and figures.

### Reporting summary

Further information on research design is available in the Nature Research Reporting Summary linked to this article.

## Supplementary information

Supplementary Information

Reporting Summary

## Data Availability

Source data are provided with this paper. The raw sequence data generated in this work were deposited into the European Nucleotide Archive (ENA) under accession identification PRJEB37442.

## Source data

Source Data

## Code availability

No custom code was used in this work.
