# Supplementary material for: Feeding diversified protein sources exacerbates hepatic insulin resistance via increased gut microbial branched-chain fatty acids and mTORC1 signaling in obese mice
Source: Nat Commun. 2021 Jun 7;12:3377. doi: 10.1038/s41467-021-23782-w (PMC8184893; doi:10.1038/s41467-021-23782-w)
Supplement: Supplementary file 1 — Supplementary Information [file 41467_2021_23782_MOESM1_ESM.pdf]

Supplementary Information

Feeding diversified protein sources exacerbates hepatic insulin resistance via increased gut microbial branched-chain fatty acids and mTORC1 signaling in obese mice

Béatrice S.-Y. Choi[#], Noëmie Daniel[#], Vanessa P. Houde, Adia Ouellette, Bruno Marcotte, Thibault V. Varin, Cécile Vors, Perrine Feutry, Olga Ilkayeva, Marcus Ståhlman, Philippe St-Pierre, Fredrik Bäckhed, Angelo Tremblay, Phillip J. White and André Marette*

# equal contribution

* Corresponding author: andre.marette@criucpq.ulaval.ca

Supplementary Figures 1-9
Supplementary Tables 1-7
Supplementary Method Tables 1-2

Supplementary Figure 1

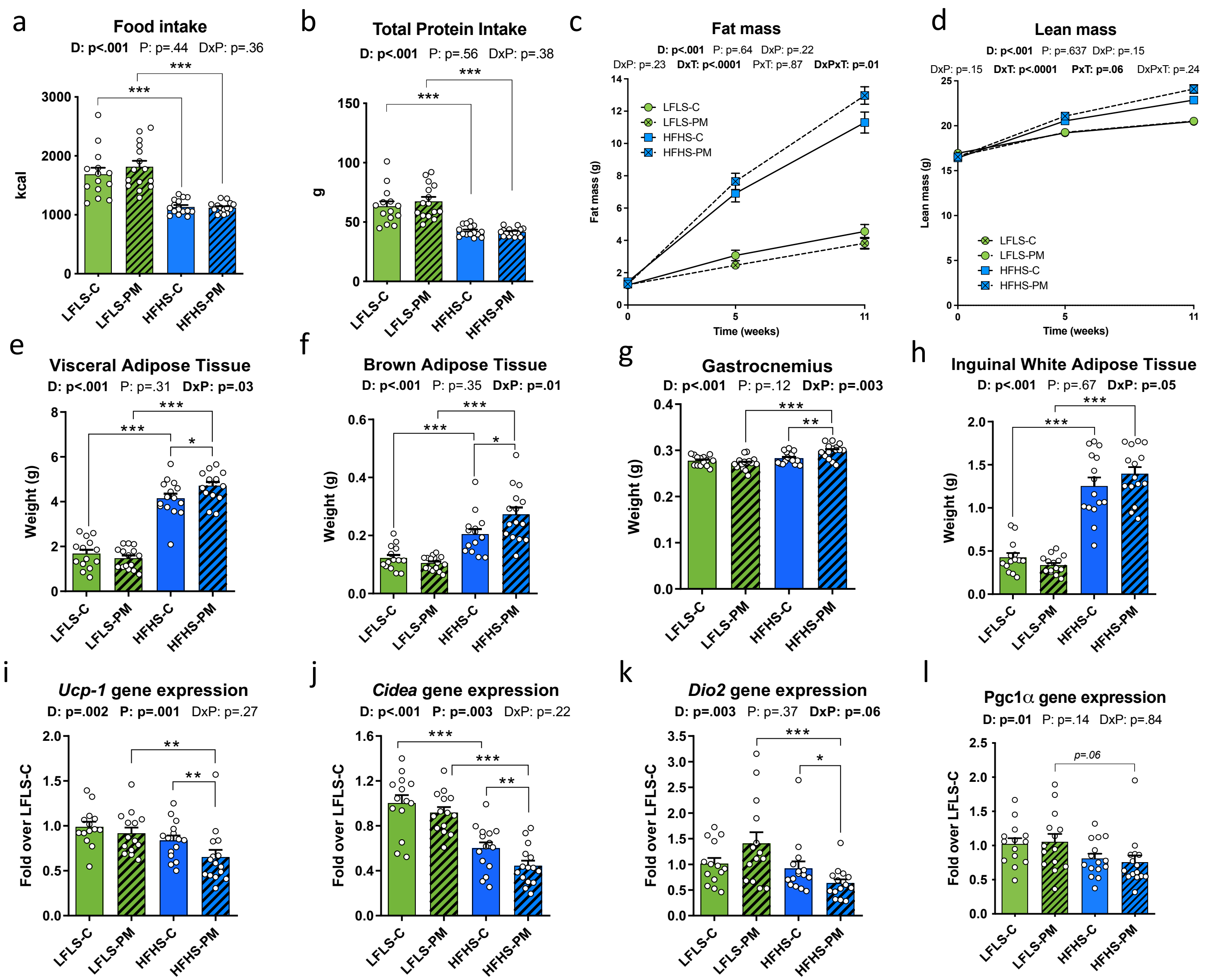

Supplementary Figure 1. Protein mix magnifies obesity-linked deleterious effect on phenotypic parameters and alters energy management. Mice were fed with a LFLS-C (green), a LFLS-PM (green, hatched), a HFHS (blue) or a HFHS-PM (blue, hatched) diet. **(a)** Total food intake and **(b)** protein intake recorded during the 12-week protocol. **(c)** Fat and **(d)** lean masses measured at weeks 0, 5 and 11 by quantitative NMR spectroscopy. **(e-h)** Tissue weights at 12 weeks of dietary treatment: **(e)** visceral adipose tissue, **(f)** intrascapular brown adipose tissue (BAT), **(f)** gastrocnemius muscle and **(h)** inguinal white adipose tissue. **(i)** *Ucp-1*, **(j)** *Cidea*, **(k)** *Dio2* and **(l)** *Pgc1α* mRNA relative gene expression quantified by RT-qPCR. Data are means±s.e.m. Statistical analyses were performed using a two-way ANOVA or a mixed model for repeated measures, followed by a Tukey post-hoc test. n=14 for LFLS-C group and n=15 biologically independent mice for the three other groups except for panels i-l where n=14 for LFLS-PM. P-values of general effect for diet (D), protein (P) and time (T) factors are recorded under the title of each graph, followed by the p-values of the corresponding factor interaction effects. Detailed significant differences detected by post-hoc test are recorded as follows: *p<.05, **p<.01, ***p<.001. Exact p-values for trends (.05 ≤ p-value < .10) are recorded on graphs for additional indication. Source data are provided as a Source Data file.

Supplementary Figure 2

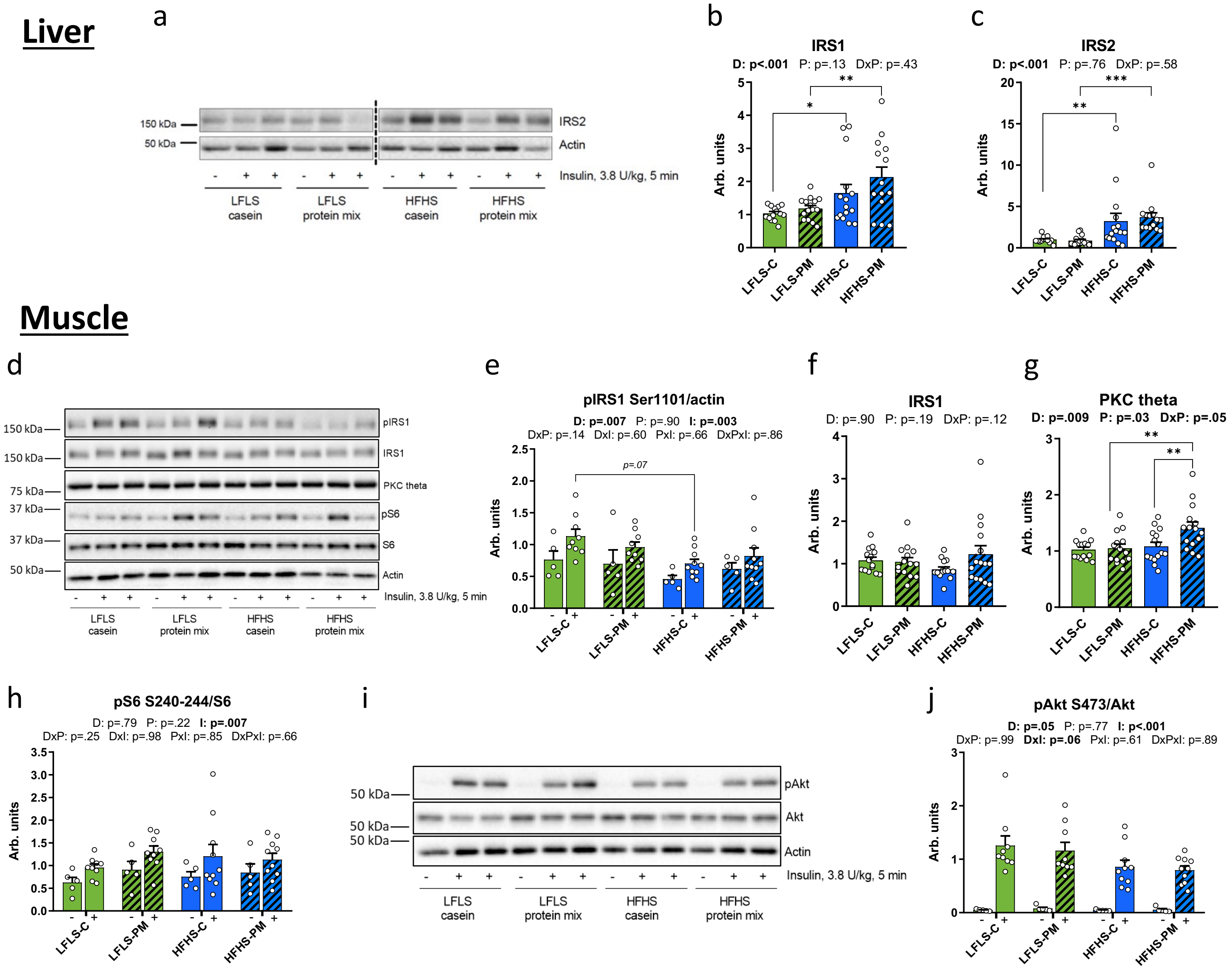

Supplementary Figure 2. Protein source does not affect total hepatic IRS1 and IRS2 and insulin signaling in the muscle. Mice were fed with a LFLS-C (green), a LFLS-PM (green, hatched), a HFHS (blue) or a HFHS-PM (blue, hatched) diet. At week 12, mice were fasted for 6 hours, injected with either saline (-) or insulin (+) and euthanized 5 minutes later. **(a-c)** Representative gels and quantification of densitometry analyses for total **(b)** IRS1 (see Figure 2 for gel) and **(a, c)** IRS2 in the liver. **(d-j)** Insulin signaling in the gastrocnemius muscle. **(d)** Representative gels and quantification of densitometry analyses for **(e)** pIRS1 Ser1101 and (**f**) total IRS1, **(g)** total PKC theta, and **(h)** pS6 S240-244. **(i)** Representative gels and quantification of densitometry analyses for **(j)** pAkt Ser473. Actin has been used as loading control. Arb. units, Arbitrary Units. Data are means±s.e.m. For total proteins (two-way ANOVA analysis), n=14-15 biologically independent mice; for insulin signaling (three-way ANOVA analysis), n=5 for all groups of independent mice injected with saline, and n=9 for LFLS-C and HFHS-PM groups and n=10 independent mice for LFLS-PM and HFHS-C groups injected with insulin. P-values of general effect for diet (D), protein (P), time (T) and insulin condition (C) factors are recorded under the title of each graph, followed by the p-values of the corresponding factor interaction effects. Detailed significant differences detected by Tukey post-hoc test are recorded as follows: *p<.05, **p<.01, ***p<.001. Exact p-values for trends (.05 ≤ p-value < .10) are recorded on graphs for additional indication. Source data are provided as a Source Data file.

Supplementary Figure 3

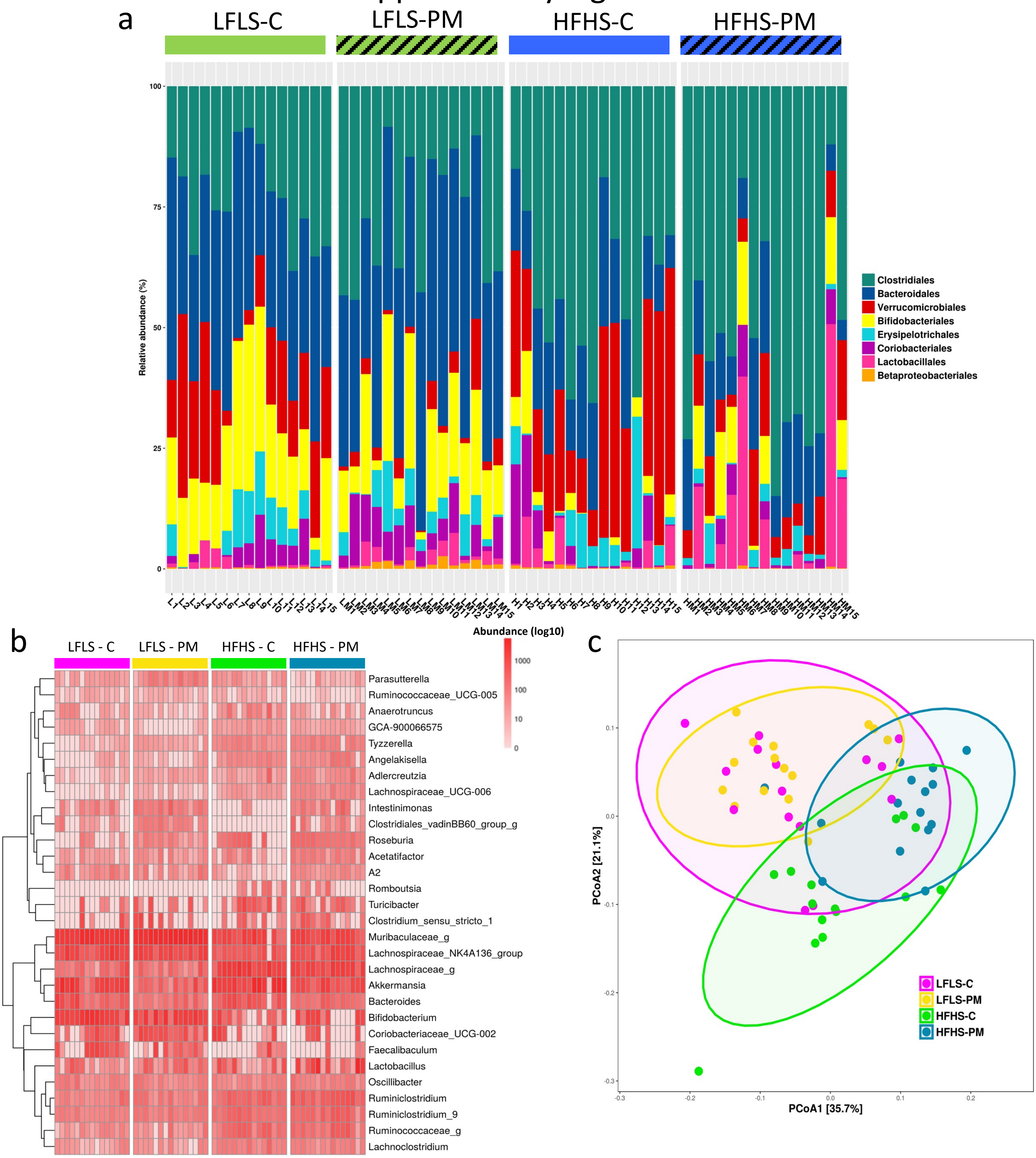

Supplementary Figure 3. Protein source and diet modulate fecal bacterial populations. (a) Stacked bar plotting the relative abundance of operational taxonomic units at order level. **(b)** Heatmap representing the overall bacterial community composition at genus level based on 16S-rRNA-encoding sequences in mice feces collected after 11 weeks of treatment. **(c)** Principal coordinates analysis (PCoA) based on unweighted Unifrac metric. The x and y axes accounted for 35.7% and 21.1% of the variance, respectively. n=14 for LFLS-C group and n=15 biologically independent mice for the three other groups. For panel a: LFLS-C (green); LFLS-PM (green, hatched); HFHS (blue);HFHS-PM (blue, hatched). **For panels b and c:** LFLS-C (pink); LFLS-PM (yellow)); HFHS (green);HFHS-PM (blue).

Supplementary Figure 4

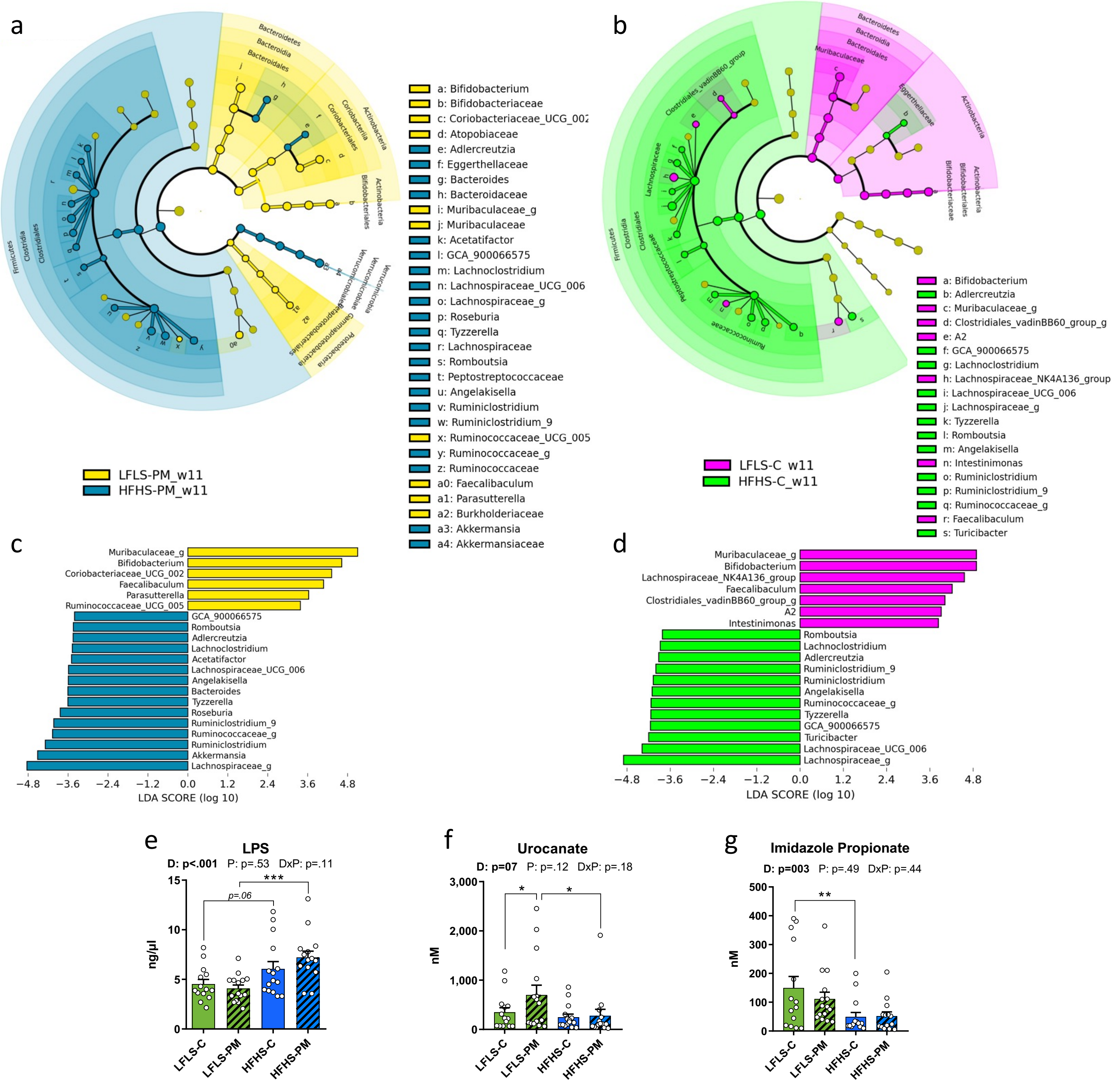

Supplementary Figure 4. HFHS diet modulates fecal bacterial populations either on casein or protein mix condition. Cladogram representations showing differentially abundant bacteria between **(a)** LFLS-PM and HFHS-PM groups and **(b)** LFLS-C and HFHS-C groups. LEfSe analysis identifying taxonomic differences between **(c)** LFLS-PM and HFHS-PM groups and **(d)** LFLS-C and HFHS-C fecal microbiotas. Histograms of LDA scores (cut-off value of 2.5) at the genus level. Plasma microbial metabolites: **(e)** Lipopolysaccharide (LPS), **(f)** urocanate and **(g)** imidazole propionate. Data are means±s.e.m. Statistical analyses were performed using a two-way ANOVA followed by a Tukey post-hoc test. n=13-15 biologically independent mice. P-values of general effect for diet (D) and protein (P) factors and diet x protein (DxP) interaction are recorded under the title of each graph. Detailed significant differences detected by post-hoc test are recorded as follows: *p<.05, **p<.01, ***p<.001. Exact p-values for trends (.05 ≤ p-value < .10) are recorded on graphs for additional indication. For panel a: LFLS-C (green); LFLS-PM (green, hatched); HFHS (blue);HFHS-PM (blue, hatched). **For panels b and c:** LFLS-C (pink); LFLS-PM (yellow); HFHS (green);HFHS-PM (blue). Source data are provided as a Source Data file.

Supplementary Figure 5

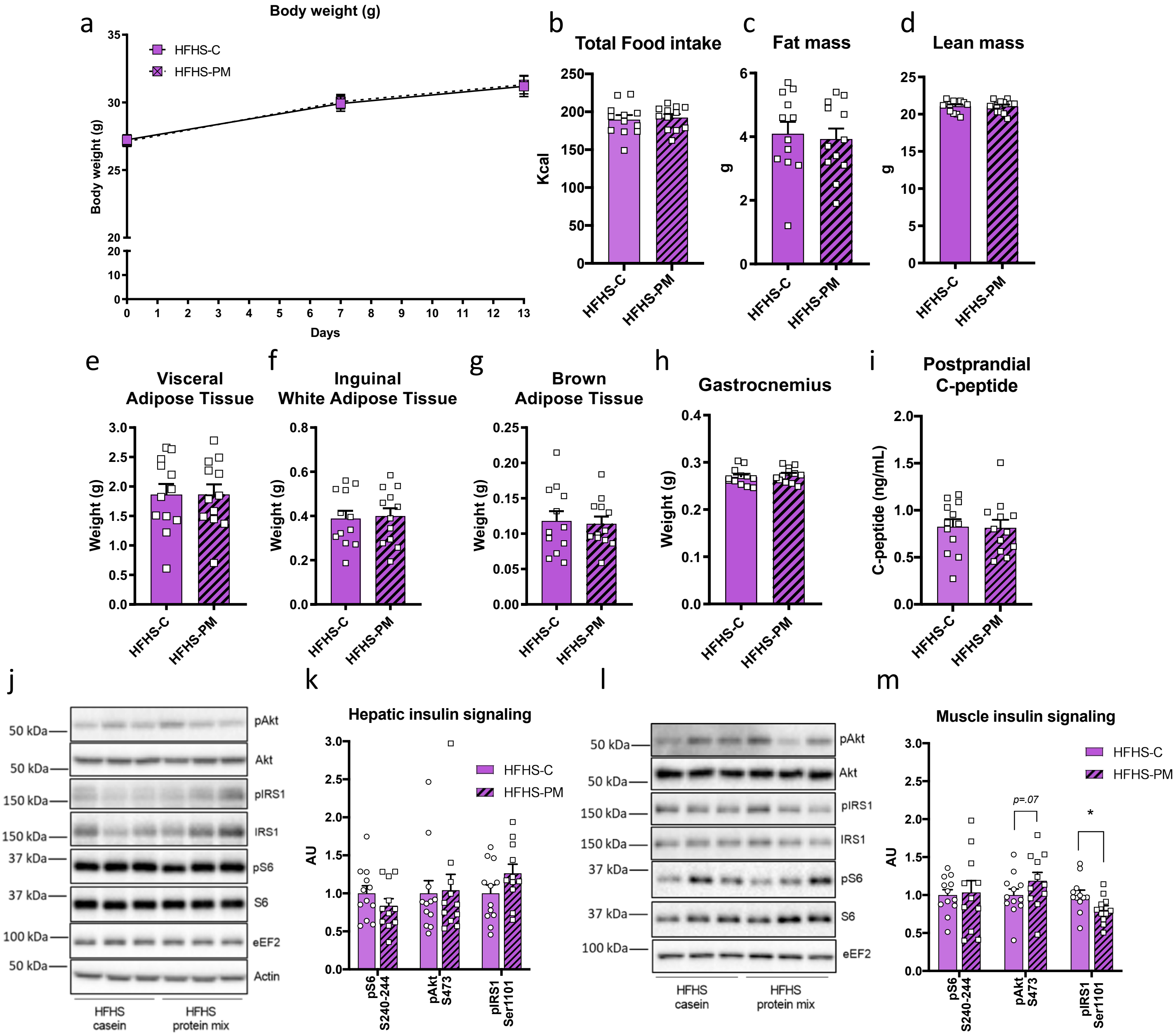

Supplementary Figure 5. Two weeks of casein replacement by protein mix is not long enough to induce metabolic or muscle insulin signaling impairment. (a) Body weight curve **(b)** total food intake, **(c)** fat mass and **(d)** lean mass measured by qNMR after 2 weeks of dietary intervention. **(e-h)** Tissue weights, (**e**) visceral adipose tissue, (**f**) inguinal white adipose tissue **(g)** intrascapular brown adipose tissue and (**h**) gastrocnemius muscle. **(i)** C-peptide in circulation in post-prandial state. n=12 independent mice for both HFHS-C (purple) and HFHS-PM (purple, hatched) groups. **(j-m)** Insulin signaling: hepatic (**k**) and muscle **(m)** quantification of densitometry analyses for pS6 S240-244 on total S6, pAkt S473 on total Akt, pIRS1 Ser1101 on total IRS1 and (**l, n**) corresponding representative immunoblots. Actin and eEF2 have been used as loading control. n=12 for HFHS-C and n=11 for HFHS-PM groups. Data are means±s.e.m. Statistical analyses were performed using a two-tailed Student's t test or its nonparametric equivalent Mann-Whitney test. Detailed significant differences are recorded as follows: *p<.05. Exact p-values for trends (.05 ≤ p-value < .10) are recorded on graphs for additional indication. Source data are provided as a Source Data file.

Supplementary Figure 6

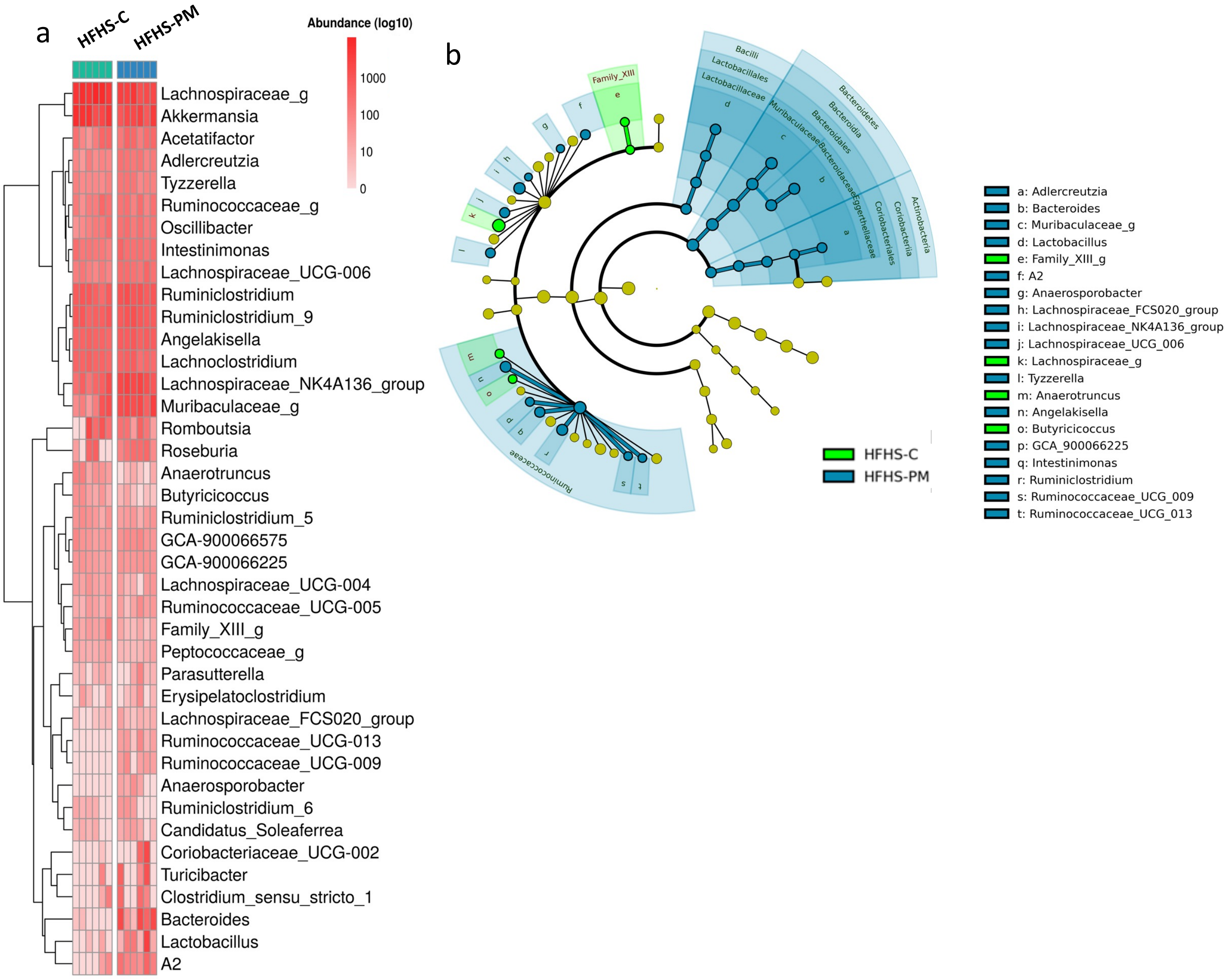

Supplementary Figure 6. Protein source modulates fecal bacteria after 2 weeks of treatment. Microbiota 16S-RNA sequencing of feces collected after 2 weeks of HFHS-C (green) or HFHS-PM (blue) dietary treatment . **(a)** Heatmap recording overall bacterial composition at the genus level. **(b)** Cladogram (the taxonomic levels from phylum to family are labelled, while genera are abbreviated). n=6 biologically independent mice for both groups. Presence of ‘g’ at the end of taxon denotes unclassified genus.

Supplementary Figure 7

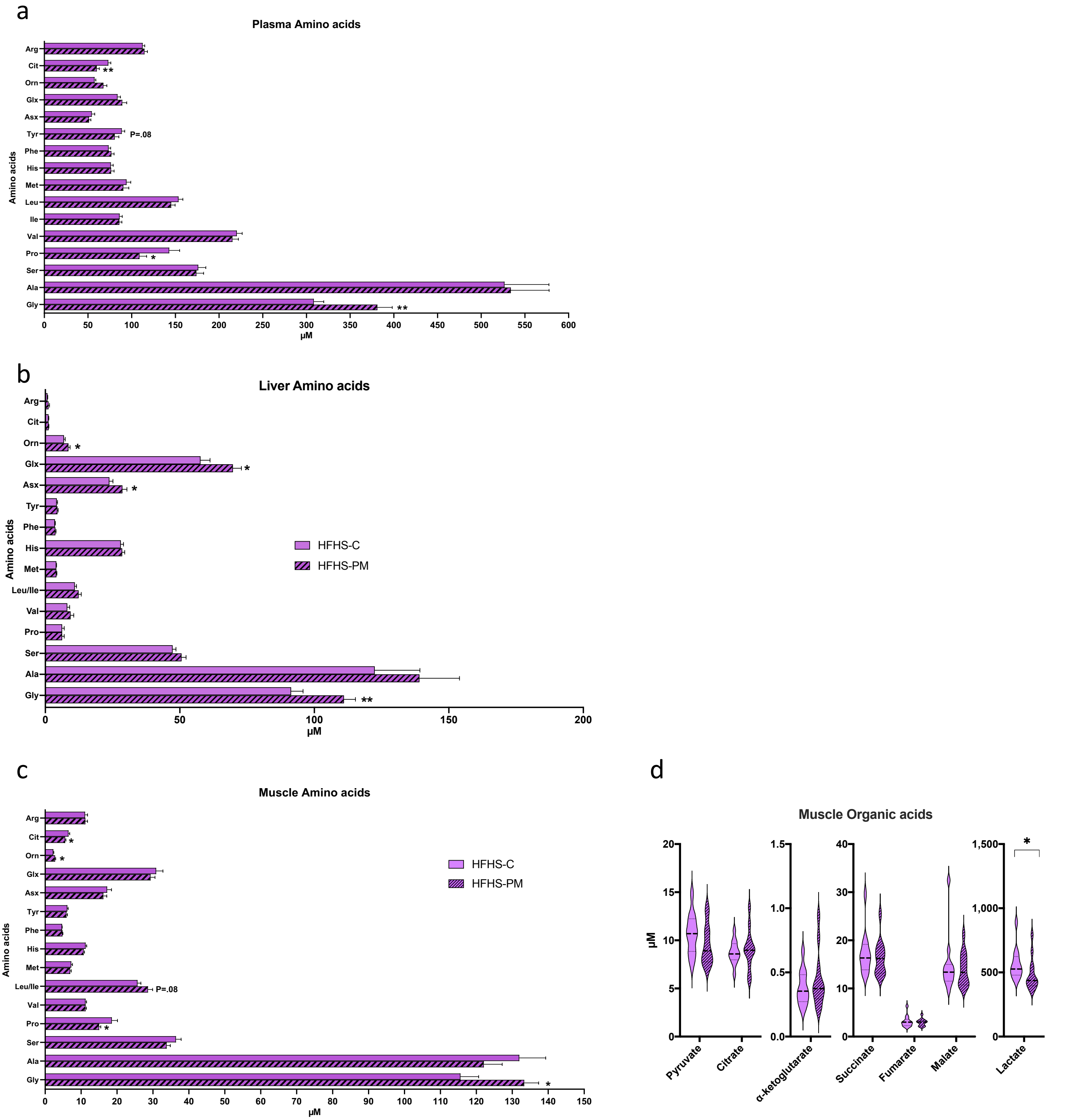

Supplementary Figure 7. Protein mix induces targeted changes in amino acids profiles in the plasma, liver and muscle of HFHS-fed mice. (a) Plasma, **(b)** liver and **(c)** muscle amino acids profiles in post-prandial state after two weeks of HFHS-C (purple) or HFHS-PM (purple, hatched) dietary challenge. **(d)** Muscle organic acids profile. n=12 independent mice for both groups. Data are means±s.e.m. Statistical analyses were performed using a two-tailed Student's t test or its nonparametric equivalent Mann-Whitney test. Detailed significant differences are recorded as follows: *p<.05, **p<.01. Exact p-values for trends (.05 ≤ p-value < .10) are recorded on graphs for additional indication. Source data are provided as a Source Data file. Arg, arginine; Cit, citrulline; Orn, ornitine; Glx, Glutamine and/or glutamate; Asx, aspartate and/or asparagine; Tyr, tyrosine; Phe, phenylalanine; Hist, histidine; Met, Methionine; Leu, leucine; Ile, Isoleucine; Val, valine; Pro, proline; Ser, serine; Ala, alanine; Gly, glycine.

Supplementary Figure 8

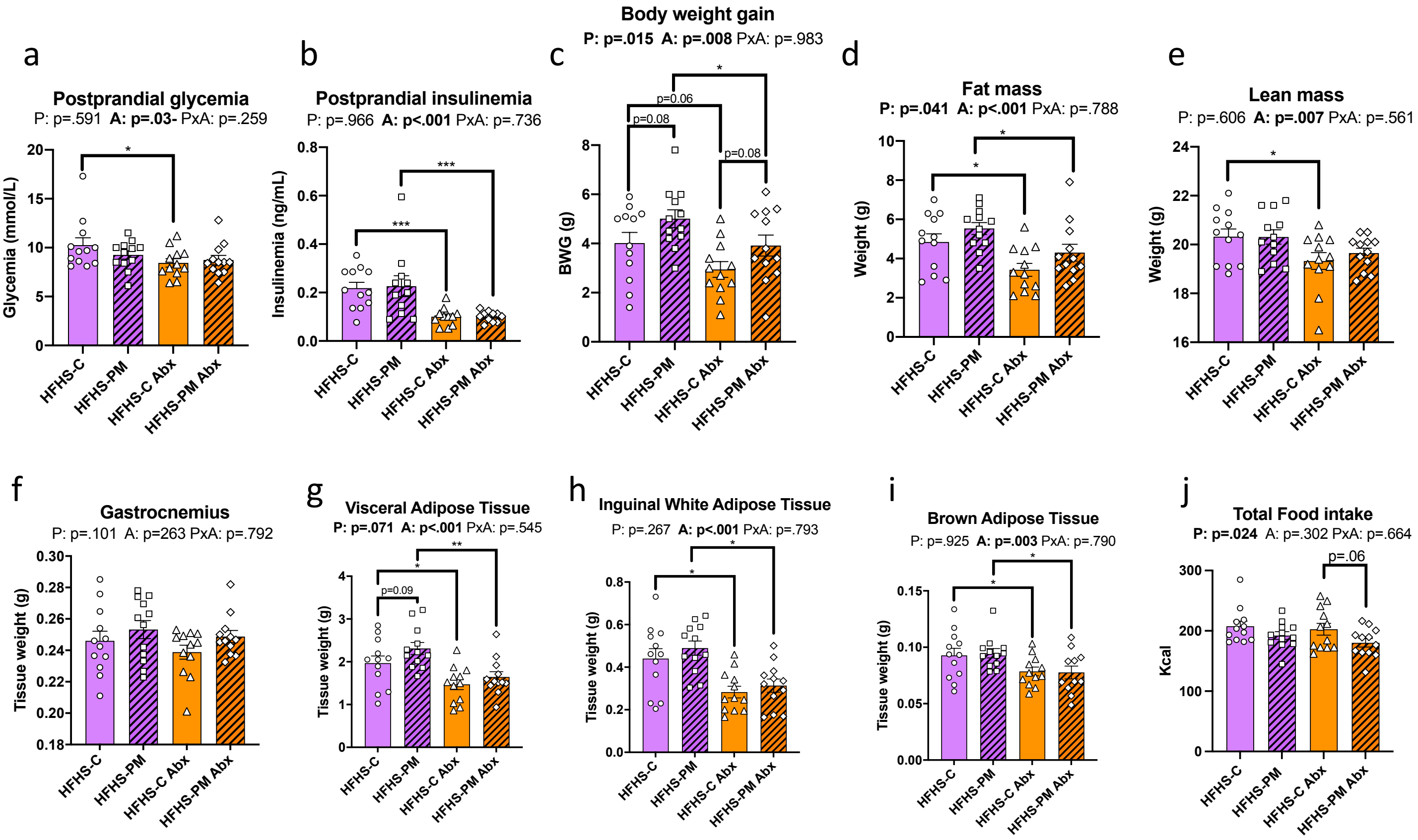

Supplementary Figure 8. Antibiotic treatment affects food intake and body composition. Mice were fed with a HFHS-C (purple) or a HFHS-PM (purple, hatched) and administered with an antibiotic cocktail (orange, and orange, hatched respectively). Physiological parameters: postprandial **(a)** glycemia **(b)** insulinemia**,** and **(c)** total body weight gain **(d)** fat mass and **(e)** lean mass measured by qNMR after 2 weeks of dietary intervention. (**f-i**) Tissue weights of (**f**) gastrocnemius muscle **(g)** visceral adipose tissue, (**h**) inguinal white adipose tissue and **(i)** intrascapular brown adipose tissue. **(j)** Total energy intake. Data are means±s.e.m. Statistical analyses were performed using a two-way ANOVA followed by a Tukey post-hoc test. n=12 biologically independent mice for all groups, except for postprandial insulinemia where n=11 for HFHS-PM, HFHS-C Abx and HFHS-PM Abx groups. P-values of general effect for protein (P) and antibiotic (A) factors and protein x antibiotic (PxA) interaction are recorded under the title of each graph. Detailed significant differences detected by post-hoc test are recorded as follows: *p<.05, **p<.01. Exact p-values for trends (.05 ≤ p-value < .10) are recorded on graphs for additional indication. Source data are provided as a Source Data file.

Supplementary Figure 9

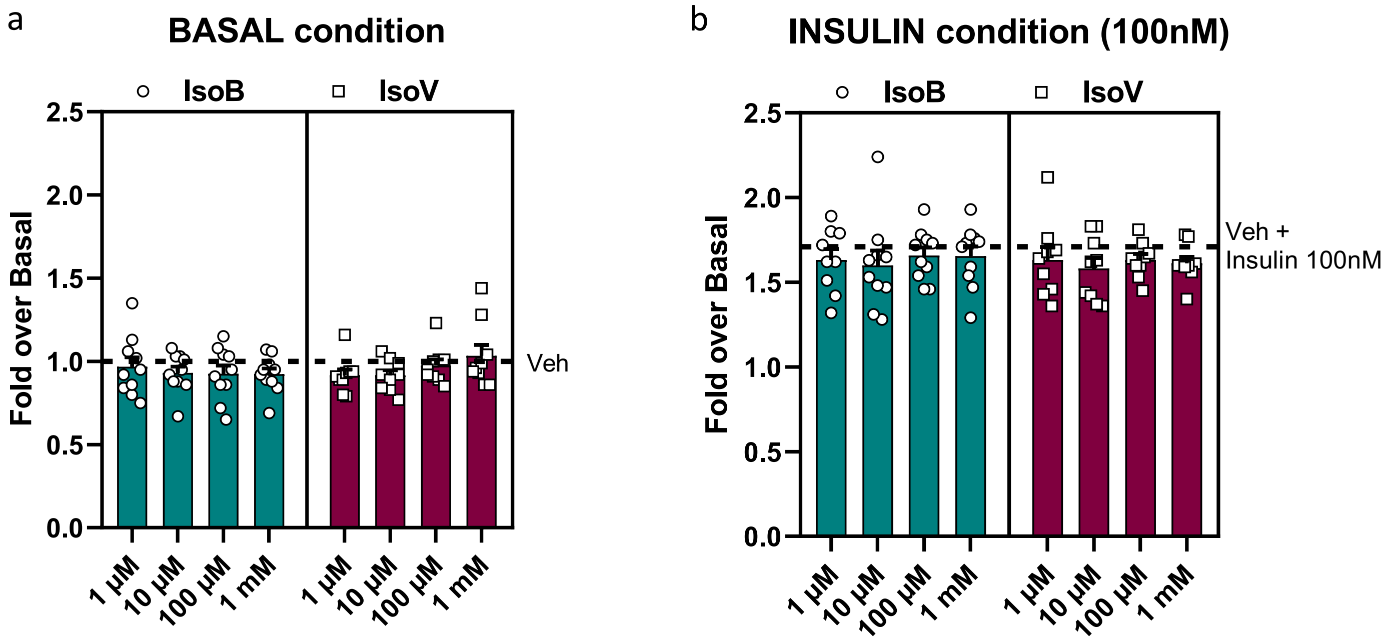

Supplementary Figure 9. BCFA do not impact glucose uptake *in vitro*. Branched chain fatty acids (BCFA) effect on skeletal muscle (L6) glucose uptake in **(a)** basal or **(b)** insulin condition (100 nM) with isobutyric (blue-green) or isovaleric acid (purple) (1-1 000µM); data corrected for total protein. Data are means±s.e.m. n=9-10 independent experiments. A Kruskal-Wallis test followed by a Dunn's post-hoc test versus Vehicule/Vehicule+Insulin, was performed in each condition. Source data are provided as a Source Data file.

SUPPLEMENTARY TABLE 1: Design of purified murine diets using a 10-source protein mix representative of human consumption.

	Dietary treatment			
Protein source	LFLS-C casein	LFLS-PM protein mix	HFHS-C casein	HFHS-PM protein mix
**Macronutrient**	**kcal %**	**kcal %**	**kcal %**	**kcal %**
**Protein**	**15**	**15**	**15**	**15**
**Carbohydrate**	**75**	**75**	**35**	**35**
sucrose	10	10	30	30
starch	65	65	5	5
**Fat**	**10**	**10**	**50**	**50**
saturated	3	3	18	18
MUFAs	3	3	15	15
PUFAs	3	3	15	14
SAT : PUFA	1	1	1	1
**Fibers (g/100g)**	**5**	**5**	**6**	**5**
**Energy content (kcal/g)**	**3.52**	**3.52**	**4.81**	**4.81**
**Ingredient**	**g**	**g**	**g**	**g**
**Protein mix**[1]	0	17.4	0	22
Protein	0	14	0	17.9
Carbohydrate	0	0.5	0	0.6
Fat	0	1.9	0	2.5
Total fibers	0	0.2	0	0.2
**Casein**	12.8	0	15.9	0
**L-cystine**	0.3	0.3	0.3	0.3
**Corn starch**[2]	62.4	68.7	5.9	6.4
**Sucrose**	6.6	7.3	30.3	34
**Cellulose**	5	5.2	5	5.3
**Lard**	2.7	1.1	16.4	16.2
**Corn oil**	1.2	1.2	7.6	8.3
**Mineral mix**[3]	6.7	6.7	6.7	6.7
**Vitamin mix**[4]	1.4	1.4	1.4	1.4
**Choline bitartrate**	0.3	0.3	0.3	0.3
**BHT**[5]	0.03	0.03	0.03	0.03
**Total grams**	99.4	109.6	89.8	100.9

[1] Home-made protein mix, see Table S1

[2] MP Biomedical corn starch : 90% starch, 10% humidity

[3] MP Biomedical mineral mixture 76 - 12% of sucrose

[4] Teklad Vitamin mix AIN-76A - 98% of sucrose

[5] Tert-butylhydroxytoluene

SUPPLEMENTARY TABLE 2: Profile of protein sources.

Protein source (Supplier, Country)		Chicken (Happy Yak, Canada)	Pork (Happy Yak, Canada)	Beef (Happy Yak, Canada)	Cod (Seagarden, Norway)	Soy (Teklad Envigo, USA)	Pea (Canadian Protein, Canada)	Rice (Canadian Protein, Canada)	Egg White (Teklad Envigo, USA)	Whey (Canadian Protein, Canada)	Casein (MP Biomedicals, USA)	Final Protein Mix (PM)
**Corresponding USDA dietary**		**Poultry**	**Meat**		**Fish**	**Legumes, fruits, nuts, soy,**		**Grains**	**Egg**	**Dairy**		
**Energy (cal/100g)**	**cal/100g**	428	451	549	389	385	399	420	322	382	363	432
**Protein**	**%**	**94.54**	**84.63**	**59.71**	**89.79**	**87.19**	**77.96**	**79.50**	**81.56**	**84.81**	**89.48**	**80.60**
**Nitrogen**	**%**	15.13	13.54	9.55	14.37	13.95	12.47	12.72	13.05	13.57	14.03	12.85
**CHO**	**%**	**0.00**	**0.00**	**0.68**	**0.00**	**2.85**	**3.56**	**7.93**	**5.72**	**4.62**	**0.23**	**2.63**
**Total dietary fiber[1]**	**g/100g**	NA	NA	NA	NA	1.70	4.80	3.20	NA	NA	NA	1.09
**Total fat**	**g/100g**	**5.51**	**12.51**	**34.19**	**3.26**	**2.81**	**8.12**	**7.80**	**0.44**	**1.35**	**0.49**	**11.06**
**SAT**	**g/100g**	1.78	4.44	15.10	0.77	0.79	1.61	3.00	0.15	0.30	0.31	4.40
**MUFA**	**g/100g**	1.84	5.36	15.39	0.61	0.42	2.13	1.96	0.17	0.27	0.10	4.33
**PUFA**	**g/100g**	1.64	2.11	1.12	1.74	1.45	4.00	2.40	0.09	0.72	0.03	1.61
**ω3**	**g/100g**	0.08	0.07	0.31	1.56	0.17	0.55	0.06	<0.01	<0.01	0.01	0.18
**ω6**	**g/100g**	1.54	1.97	0.78	0.15	1.28	3.44	2.33	0.08	0.71	0.01	1.40
**TRANS fat**	**g/100g**	0.02	0.04	1.05	0.01	0.02	0.02	0.09	<0,01	0.01	0.02	0.22
**Cholesterol**	**mg/100g**	227.60	264.70	214.70	478.30	<0.80	<0.80	<0.80	24.10	3.10	15.30	117.42
**Ash**	**%**	3.50	3.84	3.20	5.83	3.93	3.15	1.53	5.73	2.64	1.56	2.93
**Humidity**	**%**	2.13	2.32	2.22	3.64	3.21	7.17	3.24	6.51	3.59	8.22	3.85
**Proportion in protein mix (g/100g of protein mix)**		**11.9**	**12.8**	**18.2**	**3.0**	**5.7**	**6.3**	**21.5**	**3.7**	**3.7**	**13.2**	**100**
**Protein proportion in protein mix[2] (g/100g of protein)**		**13.8**	**13.4**	**13.4**	**3.3**	**6.1**	**6.1**	**21.1**	**3.7**	**3.8**	**15.4**	**100**

NA : Non Analyzed

[1] Since meats, dairy, fish, egg and casein source are not supposed to contain fibers, the total dietary fiber was calculated based on soy, pea and rice sources content.

[2] The proportion of each protein source is based on USDA data, Protein contribution from major food groups to the US food supply, 1909-2010

SUPPLEMENTARY TABLE 3: Amino acid profile of purified murine diets.

	Dietary treatment			
	LFLS-C	LFLS-PM	HFHS-C	HFHS-PM
Protein source	casein	protein mix	casein	protein mix
Amino acid profile	g/100g of diet			
BCAA	2.4	2.3	3.3	3.2
Valine	0.7	0.7	1	0.9
Isoleucine	0.6	0.6	0.8	0.8
Leucine	1.1	1.1	1.5	1.5
Aspartic acid	0.8	1.2	1.1	1.6
Threonine	0.5	0.6	0.7	0.8
Serine	0.7	0.6	0.9	0.8
Glutamic acid	2.8	2.4	3.9	3.3
Glycine	0.2	0.5	0.3	0.7
Alanine	0.3	0.6	0.5	0.9
Methionine	0.3	0.3	0.5	0.4
Tyrosine	0.6	0.5	0.9	0.7
Phenylalanine	0.6	0.7	0.8	0.9
Lysine	1.1	0.9	1.5	1.2
Histidine	0.3	0.3	0.5	0.5
Arginine	0.4	0.8	0.6	1.1
Proline	1.3	0.7	1.8	0.9
Hydroxyproline	0	0	0	0
Cysteine	0.1	0.2	0.1	0.2
Tryptophan	0.1	0.1	0.2	0.2
Others	0.2	0.1	0.2	0.1
Total	12.9	12.8	17.7	17.6

SUPPLEMENTARY TABLE 4: Plasma acylcarnitine profile in postprandial state after 2 weeks of dietary treatment. Data are means±s.e.m. Statistical analyses were performed using a two-tailed Student's t test or its nonparametric equivalent Mann-Whitney test. An FDR correction (Two-stage step-up method of Benjamini, Krieger and Yekutieli, 5%) was applied on data to correct for multiple analysis. Significant differences are bolded. n=11-12.

Plasma Acylcarnitine (AC)	HFHS-C	HFHS-PM	p-value	Adjusted FDR q-value
	µM			
**C2**	2.02E+01 ± 6.03E-01	3.09E+01 ± 2.89E+00	***0.006***	***0.011***
**C3**	2.76E-01 ± 2.82E-02	3.95E-01 ± 4.31E-02	***0.031***	*0.052*
**C4/Ci4**	3.62E-01 ± 3.26E-02	5.13E-01 ± 4.53E-02	***0.013***	***0.032***
**C4-OH (C3-DC)**	1.39E-01 ± 9.48E-03	2.16E-01 ± 3.03E-02	***0.017***	*0.046*
**C4-DC/Ci4-DC**	1.69E-02 ± 1.08E-03	2.20E-02 ± 2.09E-03	***0.041***	*0.061*
**Total short chain AC**	2.10E+01 ± 6.11E-01	3.20E+01 ± 2.97E+00	***0.005***	***0.011***
**C5**	1.14E-01 ± 8.42E-03	1.64E-01 ± 1.27E-02	***0.004***	***0.019***
**C5:1**	1.35E-01 ± 6.08E-03	1.35E-01 ± 7.00E-03	*0.996*	*0.726*
**C5-OH/C3-DC(-M)**	5.40E-02 ± 4.81E-03	5.94E-02 ± 7.92E-03	*0.908*	*0.474*
**C5-DC (C6-OH)**	1.86E-02 ± 2.10E-03	1.95E-02 ± 3.13E-03	*0.817*	*0.621*
**C6 (C4:1-DC)**	9.00E-02 ± 4.99E-03	1.46E-01 ± 1.23E-02	***<0.001***	***0.006***
**C7-DC**	2.10E-02 ± 3.70E-03	1.77E-02 ± 3.46E-03	*0.522*	*0.452*
**C8**	3.35E-02 ± 2.79E-03	4.46E-02 ± 3.77E-03	***0.027***	*0.049*
**C8:1**	5.12E-03 ± 1.43E-03	8.07E-03 ± 2.02E-03	*0.246*	*0.237*
**C8:1-DC**	6.22E-03 ± 8.48E-04	5.21E-03 ± 1.35E-03	*0.532*	*0.452*
**C8:1-OH/C6:1-DC**	2.08E-02 ± 2.50E-03	1.56E-02 ± 1.80E-03	*0.105*	*0.110*
**C6-DC/C8-OH**	3.21E-02 ± 1.40E-03	3.12E-02 ± 2.85E-03	*0.777*	*0.603*
**C10**	2.65E-02 ± 1.87E-03	4.08E-02 ± 5.40E-03	***0.004***	***0.042***
**C10-OH/C8-DC**	3.58E-02 ± 2.72E-03	4.26E-02 ± 4.12E-03	*0.182*	*0.181*
**C10:1**	2.19E-02 ± 1.31E-03	2.88E-02 ± 3.62E-03	***0.013***	*0.110*
**C10:2**	1.08E-03 ± 5.96E-04	1.73E-03 ± 6.89E-04	*0.449*	*0.431*
**C10:3**	1.08E-03 ± 6.11E-04	7.18E-04 ± 4.84E-04	*0.626*	*0.522*
**Total medium chain AC**	6.17E-01 ± 2.21E-02	7.60E-01 ± 4.47E-02	***0.009***	***0.024***
**C12**	4.55E-02 ± 3.43E-03	6.80E-02 ± 6.97E-03	***0.010***	***0.024***
**C12-OH/C10-DC**	1.39E-02 ± 1.15E-03	1.40E-02 ± 1.39E-03	*0.964*	*0.717*
**C12:1**	1.49E-02 ± 2.05E-03	2.47E-02 ± 4.12E-03	***0.045***	*0.061*
**C14**	7.90E-02 ± 3.25E-03	1.01E-01 ± 6.97E-03	***0.008***	***0.024***
**C14-OH/C12-DC**	5.99E-03 ± 1.00E-03	8.92E-03 ± 1.36E-03	*0.098*	*0.110*
**C14:1-OH**	8.17E-02 ± 5.37E-03	1.13E-01 ± 8.30E-03	***0.004***	***0.020***
**C14:1-OH**	1.62E-02 ± 1.45E-03	2.20E-02 ± 2.08E-03	***0.034***	*0.052*
**C14:2**	2.89E-02 ± 1.34E-03	3.66E-02 ± 2.64E-03	***0.016***	***0.035***
**C16**	2.54E-01 ± 1.07E-02	3.08E-01 ± 2.11E-02	***0.033***	*0.052*
**C16-OH/C14-DC**	1.03E-02 ± 9.20E-04	1.51E-02 ± 2.08E-03	***0.044***	*0.061*
**C16:1**	6.43E-02 ± 3.97E-03	7.24E-02 ± 6.54E-03	*0.303*	*0.279*
**C16:1-OH/C14:1-DC**	1.64E-02 ± 1.40E-03	2.00E-02 ± 1.58E-03	*0.102*	*0.110*
**C16:2**	2.38E-02 ± 1.72E-03	2.95E-02 ± 2.72E-03	*0.093*	*0.110*
**C18**	1.12E-01 ± 4.78E-03	1.40E-01 ± 3.35E-03	***0.001***	***0.004***
**C18-OH/C16-DC**	1.62E-02 ± 1.37E-03	2.28E-02 ± 1.38E-03	***0.003***	***0.016***
**C18:1**	2.98E-01 ± 1.09E-02	3.55E-01 ± 2.03E-02	***0.021***	***0.042***
**C18:1-DC**	8.67E-03 ± 9.18E-04	1.15E-02 ± 1.74E-03	*0.170*	*0.173*
**C18:1-OH/C16:1-DC**	1.49E-02 ± 1.07E-03	1.92E-02 ± 2.29E-03	*0.088*	*0.110*
**C18:2**	1.16E-01 ± 4.65E-03	1.43E-01 ± 8.86E-03	***0.013***	***0.032***
**C18:2-OH**	6.87E-03 ± 9.54E-04	1.06E-02 ± 8.64E-04	***0.008***	***0.024***
**C20**	7.86E-03 ± 1.14E-03	1.08E-02 ± 8.14E-04	***0.049***	*0.064*
**C20-OH/C18-DC**	1.81E-03 ± 6.19E-04	2.90E-03 ± 8.32E-04	*0.305*	*0.279*
**C20:4**	2.04E-02 ± 1.16E-03	2.50E-02 ± 2.37E-03	*0.099*	*0.110*
**C22**	5.63E-03 ± 7.26E-04	5.21E-03 ± 1.15E-03	*0.760*	*0.603*
**Total long chain AC**	1.26E+00 ± 4.70E-02	1.58E+00 ± 9.47E-02	***0.007***	***0.024***
**Total AC**	2.29E+01 ± 6.45E-01	3.44E+01 ± 3.09E+00	***0.004***	***0.011***

SUPPLEMENTARY TABLE 5 : Liver acylcarnitine profile in postprandial state after 2 weeks of dietary treatment.
Data are means±s.e.m. Statistical analyses were performed using a two-tailed Student's t test or its nonparametric equivalent Mann-Whitney test. An FDR correction (Two-stage step-up method of Benjamini, Krieger and Yekutieli, 5%) was applied on data to correct for multiple analysis. Significant differences are bolded. n=11-12.

Liver Acylcarnitine (AC)	HFHS-C	HFHS-PM	p-value	Adjusted FDR q-value
	µM			
C2	6.36E+00 ± 3.28E-01	7.96E+00 ± 4.71E-01	***0.011***	*0.195*
C3	1.74E-01 ± 2.19E-02	2.39E-01 ± 2.47E-02	*0.061*	*0.314*
C4/Ci4	2.43E-01 ± 1.50E-02	2.67E-01 ± 2.49E-02	*0.417*	*0.719*
C4-OH (C3-DC)	1.30E-01 ± 9.87E-03	1.60E-01 ± 1.73E-02	*0.141*	*0.527*
C4-DC/Ci4-DC	2.52E-02 ± 1.13E-03	2.74E-02 ± 1.77E-03	*0.299*	*0.676*
Total short chain AC	6.94E+00 ± 3.35E-01	8.66E+00 ± 4.91E-01	***0.008***	*0.195*
C5	3.58E-02 ± 2.13E-03	4.46E-02 ± 3.29E-03	***0.036***	*0.261*
C5:1	2.19E-01 ± 1.59E-02	2.19E-01 ± 1.31E-02	*0.972*	>0.999
C5-OH/C3-DC(-M)	4.36E-02 ± 2.29E-03	4.40E-02 ± 3.07E-03	*0.913*	>0.999
C5-DC (C6-OH)	1.35E-01 ± 5.51E-03	1.70E-01 ± 1.00E-02	***0.001***	*0.195*
C6 (C4:1-DC)	2.43E-02 ± 1.95E-03	2.84E-02 ± 3.53E-03	*0.707*	*0.697*
C7-DC	1.27E-01 ± 1.81E-02	1.23E-01 ± 2.74E-02	*0.470*	>0.999
C8	4.95E-02 ± 3.33E-03	8.54E-02 ± 6.83E-03	***<0.001***	***0.007***
C8:1	2.41E-03 ± 4.55E-04	1.88E-03 ± 3.43E-04	*0.436*	*0.697*
C8:1-DC	1.41E-02 ± 1.12E-03	1.72E-02 ± 1.77E-03	*0.156*	*0.527*
C8:1-OH/C6:1-DC	1.18E-02 ± 8.00E-04	1.52E-02 ± 1.83E-03	***0.026***	*0.437*
C6-DC/C8-OH	2.28E-01 ± 1.12E-02	2.45E-01 ± 2.24E-02	*0.885*	*0.797*
C10	2.03E-02 ± 1.99E-03	2.30E-02 ± 3.02E-03	*0.470*	*0.752*
C10-OH/C8-DC	1.17E-02 ± 1.58E-03	1.62E-02 ± 4.60E-03	*0.312*	*0.697*
C10:1	8.56E-03 ± 5.44E-04	9.79E-03 ± 6.45E-04	*0.160*	*0.527*
C10:2	5.44E-04 ± 2.54E-04	5.44E-04 ± 2.56E-04	*1.000*	>0.999
C10:3	1.19E-03 ± 3.75E-04	1.21E-03 ± 2.86E-04	*0.974*	>0.999
Total medium chain AC	9.33E-01 ± 3.31E-02	1.04E+00 ± 7.43E-02	*0.214*	*0.536*
C12	2.02E-02 ± 7.99E-04	2.67E-02 ± 4.12E-03	***0.040***	*0.514*
C12-OH/C10-DC	5.04E-03 ± 3.65E-04	5.64E-03 ± 5.24E-04	*0.583*	*0.697*
C12:1	7.47E-03 ± 3.43E-04	8.59E-03 ± 7.71E-04	*0.199*	*0.550*
C12:1-OH/C10:1-DC	3.39E-03 ± 1.94E-04	4.79E-03 ± 5.10E-04	***0.018***	*0.211*
C12:2	9.74E-04 ± 2.34E-04	4.06E-04 ± 1.75E-04	*0.065*	*0.314*
C12:2-OH/C10:2-DC	6.85E-03 ± 3.16E-04	6.75E-03 ± 7.58E-04	*0.907*	>0.999
C14	5.71E-02 ± 2.36E-03	6.26E-02 ± 5.47E-03	*0.366*	*0.697*
C14-OH/C12-DC	6.64E-03 ± 4.05E-04	7.94E-03 ± 1.08E-03	*0.470*	*0.675*
C14:1	4.59E-02 ± 1.73E-03	4.89E-02 ± 4.32E-03	*0.523*	*0.805*
C14:1-OH/C12:1-DC	8.24E-03 ± 6.12E-04	1.05E-02 ± 9.00E-04	***0.048***	*0.313*
C14:2	1.22E-02 ± 9.21E-04	1.32E-02 ± 1.15E-03	*0.533*	*0.805*
C14:2-OH/C12:2-DC	2.72E-03 ± 2.05E-04	3.51E-03 ± 3.32E-04	*0.055*	*0.314*
C14:3	1.16E-03 ± 1.93E-04	1.04E-03 ± 2.34E-04	*0.718*	*0.929*
C14:3-OH/C12:3-DC	1.52E-03 ± 2.93E-04	1.10E-03 ± 3.87E-04	*0.138*	*0.719*
C16	1.94E-01 ± 1.16E-02	2.01E-01 ± 1.56E-02	*0.711*	*0.929*
C16-OH/C14-DC	1.47E-02 ± 1.33E-03	1.52E-02 ± 1.22E-03	*0.796*	*0.995*
C16:1	7.67E-02 ± 4.39E-03	7.81E-02 ± 7.19E-03	*0.871*	>0.999
C16:1-OH/C14:1-DC	1.26E-02 ± 7.31E-04	1.40E-02 ± 1.01E-03	*0.261*	*0.674*
C16:2	2.31E-02 ± 2.18E-03	2.64E-02 ± 2.19E-03	*0.289*	*0.675*
C16:2-OH/C14:2-DC	5.37E-03 ± 3.57E-04	6.35E-03 ± 5.96E-04	*0.172*	*0.536*
C16:3	2.50E-03 ± 2.67E-04	2.41E-03 ± 4.47E-04	*0.860*	>0.999
C16:3-OH/C14:3-DC	5.31E-04 ± 1.69E-04	3.68E-04 ± 1.21E-04	*0.440*	*0.745*
C18	8.74E-02 ± 7.02E-03	8.78E-02 ± 5.30E-03	*0.971*	>0.999
C18-OH/C16-DC	1.05E-02 ± 8.17E-04	1.12E-02 ± 9.29E-04	*0.628*	*0.885*
C18:1	2.67E-01 ± 2.25E-02	2.62E-01 ± 2.15E-02	*0.854*	>0.999
C18:1-OH/C16:1-DC	2.05E-02 ± 1.44E-03	1.99E-02 ± 1.44E-03	*0.768*	*0.977*
C18:2	1.33E-01 ± 1.32E-02	1.33E-01 ± 1.15E-02	*0.996*	>0.999
C18:2-OH/C16:2-DC	1.28E-02 ± 8.18E-04	1.35E-02 ± 1.18E-03	*0.634*	0.885
C18:3	1.12E-02 ± 1.33E-03	1.19E-02 ± 1.03E-03	*0.670*	0.914
C18:3-OH/C16:3-DC	1.49E-03 ± 1.93E-04	2.19E-03 ± 2.04E-04	***0.021***	0.220
C20	1.85E-02 ± 2.02E-03	2.16E-02 ± 1.57E-03	*0.237*	0.635
C20-OH/C18-DC/C22:6	2.16E-03 ± 2.82E-04	3.31E-03 ± 3.92E-04	***0.027***	0.240
C20:1	2.35E-02 ± 2.09E-03	2.61E-02 ± 2.21E-03	*0.413*	0.719
C20:1-OH/C18:1-DC	4.41E-03 ± 5.51E-04	4.83E-03 ± 5.18E-04	*0.413*	0.854
C20:2	1.79E-02 ± 1.66E-03	1.80E-02 ± 1.82E-03	*0.955*	>0.999
C20:2-OH/C18:2-DC	5.86E-03 ± 4.28E-04	6.34E-03 ± 2.45E-04	*0.346*	0.697
C20:3	9.30E-03 ± 6.20E-04	8.51E-03 ± 9.30E-04	*0.491*	0.790
C20:3-OH/C18:3-DC	2.17E-03 ± 2.39E-04	1.80E-03 ± 2.36E-04	*0.280*	0.675
C20:4	1.77E-02 ± 1.25E-03	2.26E-02 ± 2.60E-03	*0.109*	0.437
C22	3.45E-03 ± 3.77E-04	3.77E-03 ± 3.97E-04	*0.563*	0.832
C22:1	6.60E-03 ± 4.53E-04	7.58E-03 ± 3.61E-04	*0.106*	0.437
C22:2	8.92E-04 ± 1.08E-04	1.08E-03 ± 1.87E-04	*0.400*	0.719
C22:3	2.85E-04 ± 9.11E-05	7.20E-04 ± 1.67E-04	***0.032***	0.261
C22:4	1.59E-03 ± 2.60E-04	2.15E-03 ± 3.11E-04	*0.181*	0.536
C22:5	4.48E-04 ± 1.54E-04	9.66E-04 ± 2.15E-04	*0.063*	0.314
Total long chain AC	1.17E+00 ± 7.47E-02	1.22E+00 ± 8.75E-02	*0.681*	0.914
Total AC	9.04E+00 ± 4.02E-01	1.09E+01 ± 6.07E-01	***0.017***	0.211

SUPPLEMENTARY TABLE 6 : Muscle acylcarnitine profile in postprandial state after 2 weeks of dietary treatment.
Data are means±s.e.m. Statistical analyses were performed using a two-tailed Student's t test or its nonparametric equivalent Mann-Whitney test. An FDR correction (Two-stage step-up method of Benjamini, Krieger and Yekutieli, 5%) was applied on data to correct for multiple analysis. Significant differences are bolded. n=11-12.

Muscle	HFHS-C	HFHS-PM		Adjusted FDR
Acylcarnitine (AC)	µM		p-value	q-value
C2	2.18E+00 ± 9.23E-02	2.46E+00 ± 1.87E-01	0.194	0.718
C3	3.58E-02 ± 4.89E-03	4.04E-02 ± 2.98E-03	0.437	0.869
C4/Ci4	6.21E-02 ± 5.46E-03	7.32E-02 ± 5.73E-03	0.172	0.718
C4-OH (C3-DC)	4.48E-02 ± 7.67E-03	7.20E-02 ± 9.18E-03	**0.033**	0.718
C4-DC/Ci4-DC	4.37E-02 ± 1.34E-03	4.31E-02 ± 2.02E-03	0.813	>0.999
Total short chain AC	2.37E+00 ± 9.83E-02	2.69E+00 ± 2.00E-01	0.163	0.718
C5	2.88E-02 ± 3.89E-03	3.02E-02 ± 4.10E-03	0.804	>0.999
C5:1	9.21E-02 ± 9.69E-03	1.12E-01 ± 5.71E-03	0.089	0.718
C5-OH/C3-DC(-M)	5.04E-02 ± 2.44E-03	4.85E-02 ± 1.54E-03	0.521	0.911
C5-DC (C6-OH)	4.21E-03 ± 1.20E-03	2.38E-03 ± 9.00E-04	0.294	0.718
C6 (C4:1-DC)	2.90E-02 ± 2.26E-03	3.08E-02 ± 3.72E-03	0.689	>0.999
C7-DC	1.93E-03 ± 6.92E-04	1.27E-03 ± 2.36E-04	0.954	0.869
C8	1.66E-02 ± 1.78E-03	1.57E-02 ± 1.65E-03	0.700	>0.999
C8:1	3.19E-03 ± 3.41E-04	2.39E-03 ± 4.61E-04	0.180	0.718
C8:1-DC	1.73E-03 ± 1.84E-04	2.27E-03 ± 5.79E-04	0.544	0.869
C8:1-OH/C6:1-DC	3.40E-03 ± 3.42E-04	3.44E-03 ± 4.04E-04	0.938	>0.999
C6-DC/C8-OH	8.23E-03 ± 7.70E-04	7.72E-03 ± 6.29E-04	0.616	0.981
C10	1.52E-02 ± 1.59E-03	1.70E-02 ± 1.51E-03	0.414	0.869
C10-OH/C8-DC	5.87E-03 ± 3.63E-04	6.22E-03 ± 6.05E-04	0.631	0.981
C10:1	3.85E-03 ± 4.82E-04	4.45E-03 ± 5.12E-04	0.406	0.869
C10:2	6.26E-04 ± 1.84E-04	1.18E-03 ± 2.83E-04	0.118	0.718
C10:3	4.46E-04 ± 1.34E-04	6.55E-04 ± 1.09E-04	0.240	0.718
Total medium chain AC	2.66E-01 ± 1.13E-02	2.86E-01 ± 1.13E-02	0.141	0.718
C12	3.27E-02 ± 3.23E-03	3.32E-02 ± 3.54E-03	0.933	>0.999
C12-OH/C10-DC	3.79E-03 ± 3.30E-04	4.74E-03 ± 4.99E-04	0.128	0.718
C12:1	8.83E-03 ± 9.58E-04	9.81E-03 ± 9.54E-04	0.480	0.896
C12:1-OH/C10:1-DC	3.75E-03 ± 5.65E-04	3.90E-03 ± 4.43E-04	0.834	>0.999
C12:2	1.36E-03 ± 2.28E-04	2.02E-03 ± 3.67E-04	0.157	0.718
C12:2-OH/C10:2-DC	5.30E-03 ± 5.18E-04	5.21E-03 ± 5.36E-04	0.907	>0.999
C14	1.14E-01 ± 1.21E-02	1.18E-01 ± 1.19E-02	0.818	>0.999
C14-OH/C12-DC	9.17E-03 ± 5.13E-04	1.05E-02 ± 1.33E-03	0.100	0.869
C14:1	4.69E-02 ± 5.42E-03	5.03E-02 ± 4.90E-03	0.640	0.981
C14:1-OH/C12:1-DC	1.25E-02 ± 7.42E-04	1.55E-02 ± 1.87E-03	0.126	0.718
C14:2	1.25E-02 ± 1.42E-03	1.30E-02 ± 1.19E-03	0.777	>0.999
C14:2-OH/C12:2-DC	3.69E-03 ± 4.10E-04	3.92E-03 ± 3.64E-04	0.682	>0.999
C14:3	8.57E-04 ± 1.97E-04	1.20E-03 ± 1.85E-04	0.215	0.718
C14:3-OH/C12:3-DC	9.19E-04 ± 2.37E-04	8.48E-04 ± 1.50E-04	0.805	>0.999
C16	6.95E-01 ± 8.29E-02	7.41E-01 ± 8.93E-02	0.712	>0.999
C16-OH/C14-DC	2.94E-02 ± 3.16E-03	3.36E-02 ± 4.67E-03	0.462	0.893
C16:1	1.08E-01 ± 9.65E-03	1.21E-01 ± 1.37E-02	0.436	0.869
C16:1-OH/C14:1-DC	1.78E-02 ± 1.36E-03	2.28E-02 ± 2.34E-03	0.081	0.718
C16:2	2.93E-02 ± 2.35E-03	3.15E-02 ± 4.01E-03	0.636	0.981
C16:2-OH/C14:2-DC	6.75E-03 ± 7.13E-04	8.90E-03 ± 1.11E-03	0.118	0.718
C16:3	2.83E-03 ± 4.09E-04	2.83E-03 ± 5.05E-04	0.996	>0.999
C16:3-OH/C14:3-DC	8.22E-04 ± 1.21E-04	8.34E-04 ± 1.66E-04	0.955	>0.999
C18	1.45E-01 ± 1.25E-02	1.64E-01 ± 1.95E-02	0.417	0.869
C18-OH/C16-DC	8.62E-03 ± 6.50E-04	1.09E-02 ± 1.13E-03	0.095	0.718
C18:1	4.09E-01 ± 3.78E-02	4.69E-01 ± 6.34E-02	0.421	0.869
C18:1-OH/C16:1-DC	2.37E-02 ± 1.79E-03	2.99E-02 ± 3.92E-03	0.165	0.718
C18:2	1.81E-01 ± 1.63E-02	2.16E-01 ± 2.93E-02	0.308	0.869
C18:2-OH/C16:2-DC	1.20E-02 ± 8.55E-04	1.63E-02 ± 2.18E-03	0.079	0.718
C18:3	9.86E-03 ± 9.16E-04	1.22E-02 ± 1.30E-03	0.147	0.718
C18:3-OH/C16:3-DC	1.21E-03 ± 1.50E-04	1.64E-03 ± 2.40E-04	0.141	0.718
C20	3.73E-03 ± 5.56E-04	4.86E-03 ± 4.40E-04	0.126	0.718
C20-OH/C18-DC/C22:6	6.13E-03 ± 1.05E-03	6.10E-03 ± 6.65E-04	0.981	>0.999
C20:1	1.96E-02 ± 1.50E-03	2.16E-02 ± 3.23E-03	0.587	0.980
C20:1-OH/C18:1-DC	2.09E-03 ± 2.10E-04	2.15E-03 ± 2.83E-04	0.872	>0.999
C20:2	1.16E-02 ± 1.47E-03	1.19E-02 ± 2.25E-03	0.905	>0.999
C20:2-OH/C18:2-DC	3.10E-03 ± 3.79E-04	3.12E-03 ± 2.63E-04	0.961	>0.999
C20:3	6.08E-03 ± 8.16E-04	5.03E-03 ± 7.53E-04	0.357	0.869
C20:3-OH/C18:3-DC	1.37E-03 ± 9.76E-05	1.12E-03 ± 1.81E-04	0.238	0.718
C20:4	1.13E-02 ± 2.15E-03	1.09E-02 ± 1.04E-03	0.883	>0.999
C22	6.47E-04 ± 1.20E-04	7.48E-04 ± 1.39E-04	0.586	0.980
C22:1	3.93E-03 ± 2.61E-04	4.29E-03 ± 1.40E-04	0.235	0.718
C22:2	1.01E-03 ± 1.56E-04	8.67E-04 ± 1.29E-04	0.489	0.896
C22:3	7.91E-04 ± 1.54E-04	6.05E-04 ± 1.36E-04	0.377	0.869
C22:4	2.76E-03 ± 4.31E-04	2.75E-03 ± 4.81E-04	0.990	>0.999
C22:5	3.75E-03 ± 6.33E-04	3.55E-03 ± 6.66E-04	0.827	>0.999
Total long chain AC	2.01E+00 ± 1.88E-01	2.23E+00 ± 2.60E-01	0.500	0.896
Total AC	4.65E+00 ± 2.18E-01	5.21E+00 ± 3.93E-01	0.224	0.718

SUPPLEMENTARY TABLE 7 : Liver acylcarnitine profile in postprandial state in the antibiotic study.

Data are means±s.e.m. Statistical analyses were performed using a two-way ANOVA followed by a Tukey post hoc test. Significant differences are bolded. n=12.

	µM				Main effects			Single effects			
Liver Acylcarnitines (AC)	HFHS-C	HFHS-PM	HFHS-C + Abx	HFHS-PM + Abx	P	A	PxA	HFHS-C vs HFHS-PM	HFHS-C+Abx vs HFHS-PM+Abx	HFHS-C vs HFHS-C+Abx	HFHS-PM vs HFHS-PM+Abx
C2	5.71E+00 ± 1.73E-01	7.17E+00 ± 2.65E-01	5.61E+00 ± 2.37E-01	6.96E+00 ± 3.61E-01	**<0.001**	0.573	0.85	**<0.001**	**0.001**	0.791	0.595
C3	1.20E-01 ± 9.13E-03	2.20E-01 ± 1.98E-02	1.71E-01 ± 2.12E-02	2.01E-01 ± 1.57E-02	**<0.001**	0.366	**0.047**	**<0.001**	0.230	**0.042**	0.428
C4/Ci4	2.21E-01 ± 1.68E-02	2.27E-01 ± 1.92E-02	1.97E-01 ± 1.84E-02	2.22E-01 ± 1.36E-02	0.364	0.407	0.579	0.801	0.302	0.329	0.845
C4-OH	1.50E-01 ± 1.05E-02	2.01E-01 ± 1.49E-02	1.37E-01 ± 2.40E-02	1.38E-01 ± 1.66E-02	0.126	**0.005**	0.197	0.049	0.861	0.256	**0.005**
C4-DC/Ci4-DC	2.50E-02 ± 2.04E-03	3.04E-02 ± 1.94E-03	2.39E-02 ± 1.74E-03	2.79E-02 ± 1.46E-03	**0.013**	0.32	0.695	**0.041**	0.128	0.668	0.328
Total SC AC	6.23E+00 ± 1.84E-01	7.85E+00 ± 2.83E-01	6.14E+00 ± 2.71E-01	7.55E+00 ± 3.83E-01	**<0.001**	0.513	0.725	**<0.001**	**0.001**	0.830	0.477
C5	5.13E-02 ± 4.49E-03	6.47E-02 ± 7.38E-03	5.56E-02 ± 3.65E-03	5.91E-02 ± 4.69E-03	0.153	0.931	0.301	0.084	0.775	0.427	0.500
C5:1	1.40E-01 ± 6.29E-03	1.31E-01 ± 6.95E-03	1.16E-01 ± 6.53E-03	1.33E-01 ± 1.09E-02	0.622	0.186	0.105	0.418	0.136	0.04	0.827
C5-OH/C3-DC	4.49E-02 ± 2.60E-03	4.87E-02 ± 3.75E-03	4.06E-02 ± 2.79E-03	3.49E-02 ± 2.01E-03	0.731	**0.003**	0.105	0.360	0.134	0.288	**0.002**
C5-DC	1.15E-01 ± 5.95E-03	1.46E-01 ± 8.04E-03	1.12E-01 ± 5.77E-03	1.36E-01 ± 8.02E-03	**<0.001**	0.367	0.596	**0.003**	**0.021**	0.791	0.312
C6	2.89E-02 ± 3.79E-03	3.25E-02 ± 6.74E-03	2.00E-02 ± 2.60E-03	1.88E-02 ± 1.35E-03	0.887	**0.003**	0.839	0.808	0.966	**0.042**	**0.022**
C7-DC	9.42E-02 ± 1.04E-02	8.77E-02 ± 6.00E-03	8.12E-02 ± 5.90E-03	8.90E-02 ± 1.19E-02	0.991	0.415	0.718	0.804	0.793	0.406	0.747
C8	6.60E-02 ± 5.97E-03	1.11E-01 ± 8.42E-03	5.05E-02 ± 3.94E-03	8.13E-02 ± 7.31E-03	**<0.001**	**0.001**	0.296	**<0.001**	**0.002**	0.104	**0.003**
C8:1	3.44E-03 ± 6.42E-04	4.14E-03 ± 1.58E-03	2.93E-03 ± 7.38E-04	3.61E-03 ± 6.40E-04	0.489	0.601	0.991	0.619	0.63	0.718	0.706
C8:1-DC	8.92E-03 ± 1.42E-03	1.15E-02 ± 2.68E-03	8.70E-03 ± 9.84E-04	1.03E-02 ± 8.55E-04	0.204	0.665	0.764	0.266	0.489	0.925	0.604
C8:1-OH/C6:1-DC	9.71E-03 ± 1.25E-03	1.31E-02 ± 1.12E-03	7.53E-03 ± 7.71E-04	1.22E-02 ± 1.00E-03	**<0.001**	0.146	0.557	**0.026**	**0.003**	0.150	0.534
C6-DC/C8-OH	1.24E-01 ± 9.18E-03	1.70E-01 ± 1.96E-02	1.33E-01 ± 2.17E-02	1.49E-01 ± 9.28E-03	**0.019**	0.65	0.621	**0.043**	0.175	0.977	0.503
C10	2.14E-02 ± 2.27E-03	2.40E-02 ± 5.56E-03	1.60E-02 ± 1.49E-03	2.06E-02 ± 2.05E-03	0.282	0.126	0.315	0.96	0.144	0.076	0.704
C10-OH/C8-DC	6.73E-03 ± 5.60E-04	8.53E-03 ± 1.11E-03	6.13E-03 ± 1.21E-03	8.14E-03 ± 9.55E-04	**0.061**	0.62	0.916	0.205	0.158	0.671	0.78
C10:1	2.43E-03 ± 6.33E-04	2.15E-03 ± 5.09E-04	4.09E-03 ± 7.22E-04	3.95E-03 ± 6.25E-04	0.741	**0.008**	0.912	0.755	0.876	0.067	**0.048**
C10:2	0.00E+00 ± 0.00E+00	0.00E+00 ± 0.00E+00	3.42E-04 ± 2.00E-04	6.66E-04 ± 3.12E-04	0.386	**0.009**	0.386	1.000	0.222	0.199	**0.015**
C10:3	2.09E-04 ± 1.53E-04	3.13E-04 ± 3.13E-04	2.55E-04 ± 2.03E-04	3.36E-04 ± 2.79E-04	0.707	0.888	0.963	0.765	0.816	0.894	0.947
Total MC AC	7.18E-01 ± 2.40E-02	8.55E-01 ± 4.64E-02	6.55E-01 ± 4.28E-02	7.61E-01 ± 3.37E-02	**0.002**	**0.044**	0.676	**0.013**	0.054	0.249	0.085
C12	2.05E-02 ± 2.97E-03	2.96E-02 ± 6.85E-03	1.49E-02 ± 3.38E-03	1.72E-02 ± 1.68E-03	0.139	**0.023**	0.881	0.343	0.248	0.083	0.125
C12-OH/C10-DC	3.22E-03 ± 5.12E-04	3.84E-03 ± 5.71E-04	3.65E-03 ± 6.78E-04	4.08E-03 ± 6.59E-04	0.395	0.589	0.876	0.477	0.623	0.623	0.786
C12:1	4.95E-03 ± 6.47E-04	6.17E-03 ± 1.13E-03	5.58E-03 ± 6.27E-04	7.14E-03 ± 7.01E-04	**0.092**	0.327	0.833	0.291	0.178	0.585	0.400
C12:1-OH/C10:1-DC	1.34E-03 ± 3.28E-04	3.28E-03 ± 6.03E-04	2.00E-03 ± 4.97E-04	2.90E-03 ± 5.29E-04	**0.007**	0.781	0.301	**0.009**	0.211	0.354	0.591
C12:2	4.79E-04 ± 2.20E-04	6.00E-04 ± 2.92E-04	0.00E+00 ± 0.00E+00	3.47E-04 ± 2.01E-04	0.268	**0.087**	0.591	0.684	0.246	0.112	0.397
C12:2-OH/C10:2-DC	2.85E-03 ± 3.92E-04	2.32E-03 ± 7.82E-04	2.80E-03 ± 4.79E-04	3.00E-03 ± 6.64E-04	0.788	0.609	0.543	0.535	0.809	0.945	0.429
C14	3.88E-02 ± 3.84E-03	5.73E-02 ± 8.70E-03	3.38E-02 ± 6.85E-03	4.00E-02 ± 3.57E-03	**0.023**	**0.047**	0.824	0.075	0.139	0.206	0.117
C14-OH/C12-DC	2.50E-03 ± 5.95E-04	3.72E-03 ± 7.98E-04	1.85E-03 ± 4.31E-04	3.21E-03 ± 6.28E-04	**0.046**	0.359	0.916	0.175	0.134	0.469	0.564
C14:1	2.28E-02 ± 2.56E-03	3.35E-02 ± 4.17E-03	2.17E-02 ± 3.00E-03	3.23E-02 ± 3.59E-03	**0.003**	0.735	0.995	**0.032**	**0.033**	0.814	0.807
C14:1-OH/C12:1-DC	3.94E-03 ± 4.08E-04	6.40E-03 ± 6.42E-04	5.02E-03 ± 7.67E-04	6.25E-03 ± 8.28E-04	**0.009**	0.499	0.375	**0.014**	0.206	0.271	0.879
C14:2	6.28E-03 ± 8.55E-04	9.78E-03 ± 1.81E-03	7.58E-03 ± 1.63E-03	1.02E-02 ± 1.02E-03	**0.027**	0.333	0.782	0.162	0.076	0.623	0.379
C14:2-OH/C12:2-DC	1.28E-03 ± 2.64E-04	2.75E-03 ± 4.53E-04	2.24E-03 ± 6.18E-04	3.31E-03 ± 6.33E-04	**0.017**	0.148	0.701	**0.049**	0.147	0.196	0.446
C14:3	1.91E-04 ± 1.91E-04	1.00E-03 ± 3.66E-04	8.24E-04 ± 5.15E-04	7.65E-04 ± 2.85E-04	0.303	0.584	0.234	0.119	0.909	0.22	0.646
C14:3-OH/C12:3-DC	1.62E-04 ± 9.59E-05	4.27E-04 ± 1.96E-04	2.94E-04 ± 2.26E-04	5.59E-04 ± 2.50E-04	0.194	0.513	1	0.357	0.357	0.644	0.644
C16	1.61E-01 ± 1.48E-02	2.09E-01 ± 2.99E-02	1.51E-01 ± 3.64E-02	1.83E-01 ± 1.70E-02	**0.038**	0.288	0.631	0.248	0.071	0.277	0.678
C16-OH/C14-DC	8.71E-03 ± 8.08E-04	1.15E-02 ± 1.66E-03	7.53E-03 ± 1.15E-03	9.76E-03 ± 1.31E-03	**0.06**	0.198	0.789	0.246	0.127	0.271	0.467
C16:1	5.22E-02 ± 6.24E-03	6.41E-02 ± 8.85E-03	4.62E-02 ± 8.75E-03	5.53E-02 ± 5.86E-03	**0.095**	0.305	0.842	0.294	0.185	0.386	0.557
C16:1-OH/C14:1-DC	6.12E-03 ± 8.13E-04	9.49E-03 ± 1.22E-03	6.53E-03 ± 9.33E-04	1.17E-02 ± 1.19E-03	**<0.001**	0.222	0.398	**0.029**	**0.001**	0.786	0.146
C16:2	1.47E-02 ± 2.06E-03	2.15E-02 ± 3.12E-03	1.47E-02 ± 2.67E-03	1.82E-02 ± 2.65E-03	**0.056**	0.549	0.75	0.114	0.252	0.842	0.517
C16:2-OH/C14:2-DC	3.56E-03 ± 3.96E-04	5.94E-03 ± 1.07E-03	3.84E-03 ± 6.90E-04	4.89E-03 ± 6.48E-04	**0.018**	0.617	0.666	**0.047**	0.161	0.961	0.510
C16:3	2.54E-03 ± 5.19E-04	3.12E-03 ± 5.30E-04	3.19E-03 ± 5.33E-04	3.18E-03 ± 5.50E-04	0.599	0.503	0.579	0.446	0.984	0.388	0.934
C16:3-OH/C14:3-DC	3.45E-04 ± 1.70E-04	6.89E-04 ± 2.36E-04	4.54E-04 ± 1.58E-04	6.42E-04 ± 2.72E-04	0.221	0.884	0.716	0.262	0.538	0.719	0.878
C18	8.64E-02 ± 6.60E-03	1.11E-01 ± 1.27E-02	8.18E-02 ± 1.13E-02	1.06E-01 ± 8.69E-03	**0.019**	0.637	0.985	0.089	0.094	0.749	0.728
C18-OH/C16-DC	7.68E-03 ± 6.02E-04	1.03E-02 ± 1.16E-03	7.98E-03 ± 8.62E-04	9.00E-03 ± 1.11E-03	**0.066**	0.616	0.419	0.063	0.455	0.827	0.356
C18:1	2.09E-01 ± 2.13E-02	2.56E-01 ± 3.28E-02	2.07E-01 ± 3.78E-02	2.50E-01 ± 2.61E-02	0.141	0.88	0.938	0.271	0.320	0.959	0.872
C18:1-OH/C16:1-DC	1.29E-02 ± 1.29E-03	1.54E-02 ± 2.07E-03	1.34E-02 ± 2.21E-03	1.62E-02 ± 2.10E-03	0.179	0.737	0.928	0.372	0.309	0.862	0.763
C18:2	9.88E-02 ± 1.10E-02	1.23E-01 ± 1.80E-02	9.52E-02 ± 2.00E-02	1.30E-01 ± 1.25E-02	**0.022**	0.989	0.396	0.287	**0.027**	0.554	0.542
C18:2-OH/C16:2-DC	1.26E-02 ± 1.37E-03	1.55E-02 ± 2.84E-03	1.22E-02 ± 1.47E-03	1.30E-02 ± 1.33E-03	0.436	0.544	0.781	0.455	0.722	0.815	0.532
C18:3	1.02E-02 ± 1.49E-03	1.25E-02 ± 2.87E-03	9.97E-03 ± 2.04E-03	1.25E-02 ± 2.28E-03	0.33	0.922	0.614	0.737	0.297	0.670	0.773
C18:3-OH/C16:3-DC	1.34E-03 ± 3.16E-04	1.13E-03 ± 3.33E-04	1.41E-03 ± 5.27E-04	1.92E-03 ± 5.04E-04	0.731	0.326	0.416	0.739	0.414	0.903	0.207
C20	1.46E-02 ± 1.43E-03	1.82E-02 ± 1.47E-03	1.61E-02 ± 2.21E-03	1.81E-02 ± 2.49E-03	0.156	0.73	0.687	0.198	0.467	0.597	0.968
C20-OH/C18-DC/C22:	1.82E-03 ± 3.81E-04	2.70E-03 ± 6.44E-04	2.51E-03 ± 3.86E-04	2.19E-03 ± 4.53E-04	0.562	0.847	0.22	0.203	0.642	0.314	0.462
C20:1	1.95E-02 ± 1.86E-03	2.27E-02 ± 2.52E-03	1.63E-02 ± 2.49E-03	1.98E-02 ± 1.85E-03	0.138	0.178	0.967	0.305	0.278	0.324	0.354
C20:1-OH/C18:1-DC	5.23E-03 ± 4.22E-04	5.86E-03 ± 5.22E-04	5.17E-03 ± 7.42E-04	6.68E-03 ± 8.09E-04	0.104	0.556	0.5	0.491	0.105	0.951	0.373
C20:2	1.20E-02 ± 1.27E-03	1.47E-02 ± 2.19E-03	1.33E-02 ± 2.07E-03	1.42E-02 ± 1.01E-03	0.294	0.791	0.6	0.267	0.708	0.577	0.854
C20:2-OH/C18:2-DC	6.47E-03 ± 3.55E-04	8.17E-03 ± 9.31E-04	5.36E-03 ± 6.18E-04	5.26E-03 ± 7.96E-04	0.265	**0.007**	0.214	0.098	0.927	0.272	**0.006**
C20:3	6.23E-03 ± 5.57E-04	8.76E-03 ± 1.16E-03	5.19E-03 ± 6.37E-04	8.59E-03 ± 8.52E-04	**<0.001**	0.473	0.604	**0.038**	**0.006**	0.383	0.888
C20:3-OH/C18:3-DC	1.36E-03 ± 4.00E-04	3.37E-03 ± 1.01E-03	1.79E-03 ± 4.64E-04	2.38E-03 ± 2.98E-04	**0.038**	0.649	0.251	**0.024**	0.493	0.621	0.258
C20:4	1.24E-02 ± 1.45E-03	2.00E-02 ± 2.66E-03	1.39E-02 ± 2.02E-03	2.63E-02 ± 2.53E-03	**<0.001**	**0.086**	0.287	**0.019**	**<0.001**	0.634	0.051
C22	2.99E-03 ± 3.10E-04	3.46E-03 ± 4.53E-04	3.70E-03 ± 4.86E-04	3.83E-03 ± 5.10E-04	0.509	0.234	0.71	0.466	0.838	0.269	0.559
C22:1	6.66E-03 ± 6.53E-04	8.05E-03 ± 1.02E-03	6.45E-03 ± 7.70E-04	6.47E-03 ± 4.99E-04	0.357	0.246	0.37	0.201	0.986	0.850	0.148
C22:2	1.15E-03 ± 2.68E-04	1.25E-03 ± 3.48E-04	7.25E-04 ± 3.69E-04	1.06E-03 ± 3.84E-04	0.538	0.378	0.728	0.850	0.496	0.385	0.705
C22:3	5.21E-04 ± 2.38E-04	6.14E-04 ± 3.74E-04	1.51E-03 ± 2.53E-04	3.16E-04 ± 1.95E-04	**0.051**	0.215	**0.023**	0.811	**0.004**	**0.014**	0.446
C22:4	1.84E-03 ± 5.08E-04	2.14E-03 ± 4.21E-04	1.58E-03 ± 4.93E-04	2.31E-03 ± 4.95E-04	0.293	0.923	0.658	0.664	0.291	0.703	0.807
C22:5	1.30E-03 ± 5.24E-04	1.15E-03 ± 3.50E-04	5.58E-04 ± 2.01E-04	1.23E-03 ± 4.24E-04	0.511	0.399	0.303	0.79	0.235	0.187	0.894
Total LC AC	8.92E-01 ± 7.38E-02	1.15E+00 ± 1.33E-01	8.58E-01 ± 1.47E-01	1.08E+00 ± 8.78E-02	**0.017**	0.507	0.745	0.135	0.054	0.485	0.811
Total AC	7.84E+00 ± 2.41E-01	9.85E+00 ± 4.07E-01	7.65E+00 ± 4.28E-01	9.39E+00 ± 4.56E-01	**<0.001**	0.412	0.722	**<0.001**	**0.003**	0.742	0.406

Supplementary Method Table 1: Antibodies and immunoblotting conditions

Antibody	Company	Catalogue number	Blocking reagent	Diluent (in TBST)	Dilution
Akt	Cell Signaling Technology	9272	BSA 5%	BSA 5%	1:2000
Phospho-Akt S473	Cell Signaling Technology	9271	BSA 5%	BSA 5%	1:2000
IRS1	Millipore	06-248	BSA 5%	BSA 5%	1:1000
IRS2	Millipore	06-506	BSA 5%	BSA 5%	1:1000
Phospho-IRS1 S1101	Cell Signaling Technology	2388	BSA 5%	BSA 5%	1:1000
Phospho-S6 S240-244	Cell Signaling Technology	5364	BSA 5%	BSA 5%	1:10000
S6	Cell Signaling Technology	2217	BSA 5%	BSA 5%	1:10000
PKC theta	Cell Signaling Technology	13643	BSA 5%	BSA 5%	1:1000
eEF2	Cell Signaling Technology	2332	BSA 5%	BSA 5%	1:1000
Actin	Millipore	MAB1501	Milk 5%	Milk 5%	1:20000
Anti-rabbit IgG-HRP	Jackson ImmunoResearch	111-035-144		Milk 5%	1:10000
Anti-mouse IgG-HRP	Jackson ImmunoResearch	115-035-146		Milk 5%	1:10000

Supplementary Method Table 2: Primer sequences

Gene	Forward	Reverse
Ucp1	ACTGCCACACCTCCAGTCATT	CTTTGCCTCACTCAGGATTGG
Cidea	TGCTCTTCTGTATCGCCCA	GCCGTGTTAAGGAATCTGCTG
PGC1α	TGGATGAAGACGGATTGC	TGGTTCTGAGTGCTAAGAC
Dio2	CAGTGTGGTGCACGTCTCCAATC	TGAACCAAAGTTGACCACCAG
Actb	CTCTAGACTTCGAGCAGGAG	AGAGTACTTGCGCTCAGGAG
Hprt	CCCCAAAATGGTTAAGGTTGC	AACAAAGTCTGGCCTGTATCC
Akkermansia muciniphila	CAGCACGTGAAGGTGGGGAC	CCTTGCGGTTGGCTTCAGAT
