# Supplementary material for: Feeding diversified protein sources exacerbates hepatic insulin resistance via increased gut microbial branched-chain fatty acids and mTORC1 signaling in obese mice
Source: Nat Commun. 2021 Jun 7;12:3377. doi: 10.1038/s41467-021-23782-w (PMC8184893; doi:10.1038/s41467-021-23782-w)
Supplement: Supplementary file 2 — Reporting Summary [file 41467_2021_23782_MOESM2_ESM.pdf]

nature research

Corresponding author(s): André Marette

Last updated by author(s): Mar 30, 2021

Reporting Summary

Nature Research wishes to improve the reproducibility of the work that we publish. This form provides structure for consistency and transparency in reporting. For further information on Nature Research policies, see Authors & Referees and the Editorial Policy Checklist .

Statistics

For all statistical analyses, confirm that the following items are present in the figure legend, table legend, main text, or Methods section.

n/a	Confirmed	
☐	☒	The exact sample size (*n*) for each experimental group/condition, given as a discrete number and unit of measurement
☐	☒	A statement on whether measurements were taken from distinct samples or whether the same sample was measured repeatedly
☐	☒	The statistical test(s) used AND whether they are one- or two-sided<br>*Only common tests should be described solely by name; describe more complex techniques in the Methods section.*
☐	☒	A description of all covariates tested
☐	☒	A description of any assumptions or corrections, such as tests of normality and adjustment for multiple comparisons
☐	☒	A full description of the statistical parameters including central tendency (e.g. means) or other basic estimates (e.g. regression coefficient) AND variation (e.g. standard deviation) or associated estimates of uncertainty (e.g. confidence intervals)
☐	☒	For null hypothesis testing, the test statistic (e.g. *F*, *t*, *r*) with confidence intervals, effect sizes, degrees of freedom and *P* value noted<br>*Give P values as exact values whenever suitable.*
☒	☐	For Bayesian analysis, information on the choice of priors and Markov chain Monte Carlo settings
☒	☐	For hierarchical and complex designs, identification of the appropriate level for tests and full reporting of outcomes
☒	☐	Estimates of effect sizes (e.g. Cohen's *d*, Pearson's *r*), indicating how they were calculated

Our web collection on statistics for biologists contains articles on many of the points above.

Software and code

Policy information about availability of computer code

Data collection

No custom code or software has been developed and applied to collect data in current study. Excel and Graphpad Prism software were used to compile data and make graphs and figures.

Data analysis

SigmaPlot, San Jose, CA, USA v12.0
Microsoft Excel v16.47.1
GraphPad Prism v.9.1.0
R (http://www.R-project.org) v3.6.0
Packages in R :
- Cutadapt v3.1
- DADA2 package v1.14.0
- RDP classifier algorithm v2.2
- DECIPHER v2.14.0
- Phanforn v.2.5.5
-Phyloseq v1.28
Image J v.1.52
SAS Studio, SAS® University Edition USA

All the softwares and codes used in current study are either commercial or have been previously published. Detailed information are provided in the methods. No custom code has been developed and applied to analyze data in current study. For the SAS code used for mixed model, see below :

Code and Software – Statistical analysis for parameters involving repeated measures
Software : SAS
Version : SAS Studio, SAS® University Edition USA

```
Code used for the variable "body weight" (as an example):
proc mixed data=proteines_log;
class nomat diet prot time;
model weight=diet|prot|time/DDFM=kenwardroger vciry solution outpm=residu;
repeated time /subject=nomat type=ar(1);
lsmeans diet*prot*time/pdiff adjust=tukey;
run;
```

The same code was used for the following variables: anthropometry (fat mass, lean mass) and glucose tolerance test (glycemia, insulinemia).

For manuscripts utilizing custom algorithms or software that are central to the research but not yet described in published literature, software must be made available to editors/reviewers. We strongly encourage code deposition in a community repository (e.g. GitHub). See the Nature Research guidelines for submitting code & software for further information.

Data

Policy information about availability of data

All manuscripts must include a data availability statement. This statement should provide the following information, where applicable:

- Accession codes, unique identifiers, or web links for publicly available datasets
- A list of figures that have associated raw data
- A description of any restrictions on data availability

The datasets generated during and/or analysed during the current study are available from the corresponding author on reasonable request.
All raw sequences were deposited in the public European Nucleotide Archive server under accession number PRJEB37442.
Silva database (formated for DADA2) was used : https://zenodo.org/record/1172783#.YGEfARKQikA

Field-specific reporting

Please select the one below that is the best fit for your research. If you are not sure, read the appropriate sections before making your selection.

☒ Life sciences ☐ Behavioural & social sciences ☐ Ecological, evolutionary & environmental sciences

For a reference copy of the document with all sections, see nature.com/documents/nr-reporting-summary-flat.pdf

Life sciences study design

All studies must disclose on these points even when the disclosure is negative.

Sample size	No sample-size calculation was performed. Sample sizes were chosen according to previous protocols performed in the laboratory.<br>DOI: 10.1093/jn/nxaa217<br>DOI: 10.1136/gutjnl-2017-315565
Data exclusions	In the 12-week study, a mouse has a much lower body weight upon arrival and was excluded for all parameters, which explains why there is only 14 mice in the group LFLS-C group. During the GF study, a problem occurred with gavage of glucose for one mice in the FMT HFHS-PM group and the animal was excluded for all values related to the OGTT. No other data were purposely excluded, other missing values are due to experimental issues.
Replication	None of the animal studies were replicated per se, although some presented in the manuscript have similar features. For cell studies, experiments in L6 were replicated 10 times (in triplicates) and in FAO 5-16 times (in duplicates and if standard deviation was above 0.2, the condition was excluded for a specific experiment).
Randomization	Mice were allocated in the different groups as they were matched for their initial body weight before the treatment start.
Blinding	Blinding was not possible during data collection as the students who allocated mice in groups were also in charge of data collection. However, measurements of metabolites such as SCFA, BCFA, acylcarnitines, amino acids, organic acids, imidazole prpopionate, urocanate were done blindly as well as gut microbiota analysis.

Reporting for specific materials, systems and methods

We require information from authors about some types of materials, experimental systems and methods used in many studies. Here, indicate whether each material, system or method listed is relevant to your study. If you are not sure if a list item applies to your research, read the appropriate section before selecting a response.

Materials & experimental systems

n/a	Involved in the study	
☐	☒	Antibodies
☐	☒	Eukaryotic cell lines
☒	☐	Palaeontology
☐	☒	Animals and other organisms
☒	☐	Human research participants
☒	☐	Clinical data

Methods

n/a	Involved in the study	
☒	☐	ChIP-seq
☒	☐	Flow cytometry
☒	☐	MRI-based neuroimaging

Antibodies

Antibodies used

Akt: Cell Signaling Technology, 9272
Phospho-Akt S473: Cell Signaling Technology, 9271
Insulin receptor beta: Cell Signaling Technology, 3025, clone 4B8
IRS1: Millipore, 06-248
IRS2: Millipore, 06-506
Phospho-IRS1 S1101: Cell Signaling Technology, 2385
PhosphoS6 S240-244: Cell Signaling Technology, 5364, clone D68F8
S6: Cell Signaling Technology, 2217, clone 5G10
PKC theta: Cell Signaling Technology, 13643, clone E1I7Y
eEF2: Cell Signaling Technology, 2332
Actin: Millipore, MAB1501, clone C4
Anti-rabbit IgG-HRP: Jackson ImmunoResearch, 111-035-144
Anti-mouse IgG-HRP: Jackson ImmunoResearch, 115-035-146

Validation

The specificity of the antibodies has been validated by the manufacturers (Cell Signaling).

Eukaryotic cell lines

Policy information about cell lines

Cell line source(s)

L6 cells (rat myoblast): gift from Amira Klip, Hospital for Sick Children, Toronto (Biochem J. 1987 Feb 15; 242(1): 131–136.) Originally from ATCC.
FAO cells (rat hepatoma): gift from Ronald Kahn, Harvard (Endocrinology 113:1201-1209, 1983), origimally from Sigma Aldrich.

Authentication

No authentication was performed on cell lines.

Mycoplasma contamination

Cell lines were tested for mycoplasma contamination periodically and all cell lines tested negative.

Commonly misidentified lines
(See ICLAC register)

No commonly misidentified cell lines were used in the study.

Animals and other organisms

Policy information about studies involving animals; ARRIVE guidelines recommended for reporting animal research

Laboratory animals

C57bl/6J male mice were used for all animal studies.
Age when treatments started :
12-week study : 8 weeks
2-week study : 10 weeks
GF study : 7-9 weeks
Abx study : 11 weeks

Wild animals

The study did not involve wild animals.

Field-collected samples

The study did not involve samples collected from the fiels.

Ethics oversight

The study was approved by the Comité de protection des animaux de l'Université Laval (Université Laval Animal Protection Committee) Approval #2017-156-1

Note that full information on the approval of the study protocol must also be provided in the manuscript.
